# The AML-Associated A338V Mutation Rewires GPR183 Signaling through C‑Terminal Ensemble Redistribution

**DOI:** 10.1021/jacs.6c10287

**Published:** 2026-10-08

**Authors:** Louise Andersson, Patryk A. Wesołowski, Laura Jahrstorfer, Alexander De Rosa, Tomáš Heger, Vilmos Neuman, Adam K. Sieradzan, David J. Wales, Paweł Kozielewicz

**Affiliations:** † Molecular Pharmacology of GPCRs, Department of Physiology and Pharmacology, 27106 Karolinska Institutet, Solna, Stockholm 171 65, Sweden; ‡ Yusuf Hamied Department of Chemistry, 2152 University of Cambridge, Lensfield Road, Cambridge CB2 1EW, United Kingdom; § Department of Chemistry, Physical and Theoretical Chemistry Laboratory, University of Oxford, South Parks Road, Oxford OX1 3QZ, United Kingdom; ∥ Department of Theoretical Chemistry, University of Gdansk, Wita Stwosza 63, Gdansk 80-308, Poland; ⊥ Samsung Medical Center, Sungkyunkwan University School of Medicine, 81, Irwon-ro, Gangnam-gu, Seoul 06351, Korea

## Abstract

G protein–coupled receptors (GPCRs) rely on dynamic conformational ensembles to coordinate G protein activation and recruitment of regulatory transducers such as GPCR kinases and β-arrestins. The oxysterol receptor GPR183 has been implicated in hematological malignancies, including acute myeloid leukemia (AML), where a rare A338V substitution occurs within its intrinsically disordered C-terminal tail. Here, we combined bioluminescence/fluorescence resonance energy transfer, bioluminescence- and fluorescence-based assays as well as migration experiments, with molecular dynamics simulations and allosteric communication networks to define the structural and functional consequences of this mutation. The A338V variant preserved agonist potency and largely maintained or even enhanced agonist-induced Gi protein activation, likely by inducing different receptor core conformations. On the other hand, it consistently impaired GRK2 and β-arrestin2 recruitment across multiple experimental configurations. Atomistic simulations supported these observations and further suggested that A338V reshapes the conformational ensemble of the C-terminal tail, altering its disorder, compaction, and membrane interactions. In particular, the mutation redistributes lipid interactions of phosphorylation-prone serine and threonine residues within the tail, suggesting disrupted spatial presentation of regulatory motifs required for productive GRK2 and β-arrestin coupling. Importantly, overexpression of GPR183 A338V in differentiated HL-60 cells led to a significant increase in migration of these cells toward the chemoattractant. Together, these results identify the intrinsically disordered C-terminal tail of GPR183 as a determinant of selective transducer coupling and show how a single naturally occurring mutation within a GPCR intrinsically disordered region can alter its conformational ensemble, selectively modulate intracellular protein coupling, and affect receptor-mediated cellular behavior.

## Graphical abstract

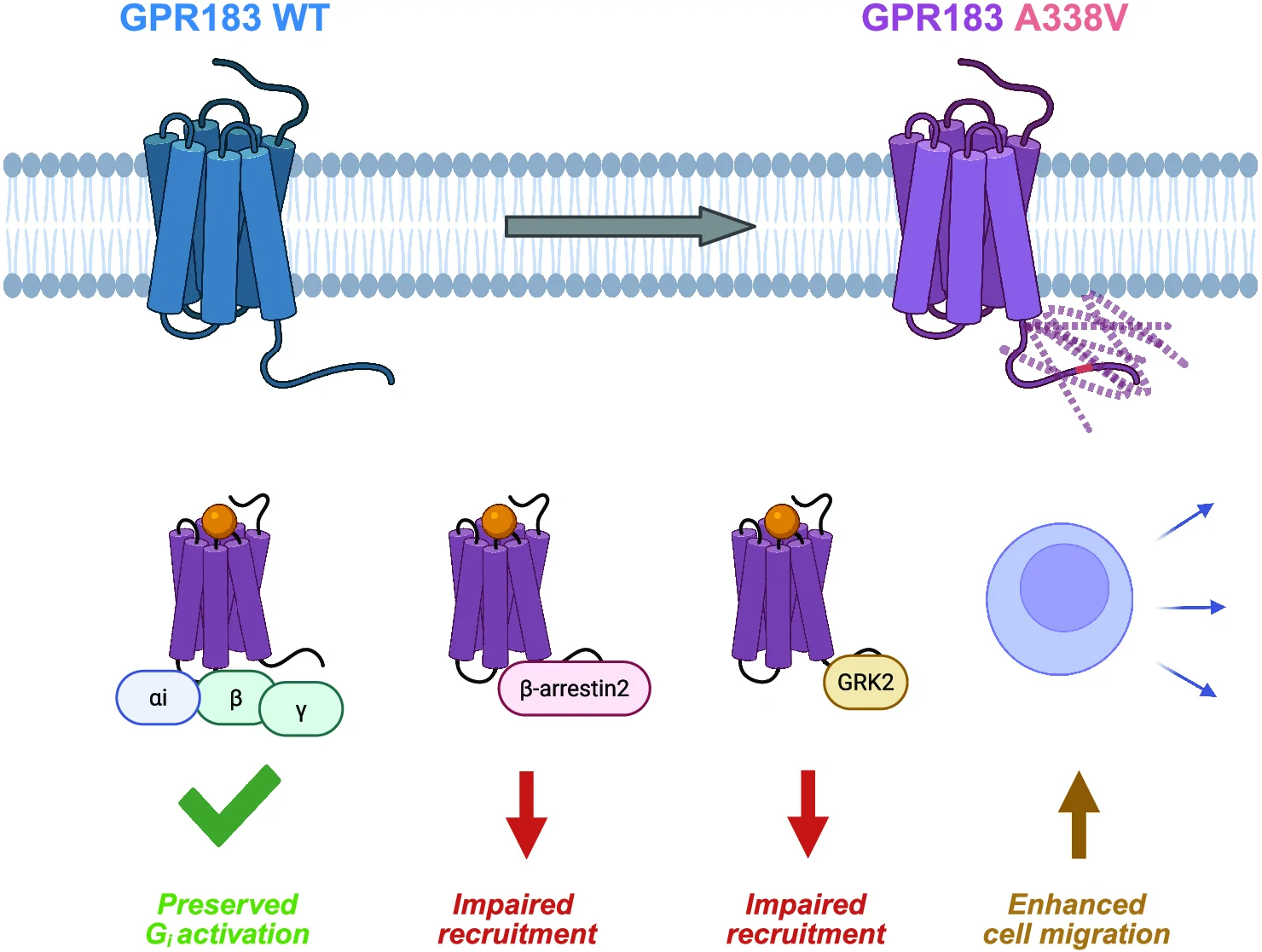

## Introduction

G protein-coupled receptors (GPCRs) constitute the largest family of membrane proteins involved in signal transduction and represent major pharmacological targets.^1^ Their functional importance reflects a central role in translating extracellular stimuli into intracellular responses through conserved conformational rearrangements within the seven transmembrane helices.^2,3^ GPR183 (EBI2) is a Class A orphan GPCR that regulates immune cell migration in response to oxysterol gradients.^4,5^ GPR183-mediated migration depends on both Gi protein activation and β-arrestin recruitment.^6^ Notably, the functional consequences of GPR183 expression and activation in pathophysiological settings are context-dependent: the receptor has been associated with beneficial roles in Burkitt lymphoma^7^ yet it appears to have detrimental effects in acute myeloid leukemia (AML).^8^ A missense substitution of alanine with valine at position 338 (A338V) within the GPR183 C-terminal tail (C-tail) has been identified as a rare AML-associated variant through genomic sequencing efforts.^9–11^ The A338V substitution occurs within the intrinsically disordered C-terminal tail of GPR183. GPCR C-terminal intrinsically disordered regions (IDRs) are highly dynamic regulatory elements that coordinate phosphorylation, membrane interactions, receptor desensitization, trafficking, and β-arrestin recruitment through heterogeneous conformational ensembles rather than fixed structures. Consequently, subtle sequence changes within these regions may redistribute conformational sampling, selectively alter transducer coupling without substantially perturbing the receptor core and encode intrinsically multifunctional capabilities.^6,12–14^

Advances in structural biology have revealed the molecular mechanisms of GPCRs by resolving high-resolution structures of many receptors bound to diverse ligands and signaling partners.^15^ The advent of AlphaFold has further revolutionized the field by enabling accurate structural predictions across nearly all GPCRs.^16^ Nevertheless, GPCRs function as dynamic ensembles rather than static entities. Their conformational landscapes underpin ligand selectivity and the formation of active states required for recognition and coupling to heterotrimeric G proteins, β-arrestins, and GPCR kinases (GRKs). These conformational states exhibit distinct lifetimes and interconversion probabilities that are modulated by cellular and environmental contexts. Capturing GPCR functional complexity, including transducer specificity, biased signaling, and allosteric regulation, therefore requires integrating static structural snapshots with insights into receptor and transducer dynamics.^17^

IDRs, notably intracellular loops and C-terminal tails, further fine-tune GPCR signaling.^18^ These regions are highly flexible and structurally heterogeneous but they are often truncated or unresolved in X-ray or cryo-EM structures due to insufficient electron density.^19^ Likewise, current data driven structure predictions tools cannot describe the full conformational ensembles of highly dynamic IDRs. Along these lines, the structural and regulatory consequences of the GPR183 A338V mutation, which occurs within this dynamic C-tail region, remain undefined. Given the sensitivity of GPCR regulatory mechanisms to local structural modifications, alterations within the C-tail may affect receptor stability and transducer interactions, as previously observed for several GPR183 C-tail deletions.^6^ We therefore hypothesized that the A338V substitution alters the conformational dynamics of the GPR183 C-terminal tail, thereby modulating proximal signaling events, including G protein activation, GRK and β-arrestin recruitment, as well as potentially affecting more distant pathways, relative to the wild-type (WT) receptor. These features all agree with the intrinsically multifunctional hypothesis.^20,21^ We used complementary experimental approaches, including G protein activation measurements, β-arrestin2 and GRK2 recruitment assays, assessment of alterations in receptor conformation, and molecular dynamics simulations to evaluate the mutation’s impact on receptor structure and function. Specifically, we combined wet lab bioluminescence and fluorescence resonance energy transfer (BRET, FRET) assays, fluorescence assays, internalization and migration experiments with AlphaFold-derived models of unresolved IDRs, including the C-tail region harboring the A338V mutation, followed by molecular dynamics simulations in explicit lipid bilayers and solvent to probe their structural dynamics and functional consequences. Importantly, we developed a cpGFP-based conformational biosensor for GPR183 that preserves the unmodified C-terminal tail, providing an independent readout of mutation-dependent changes in receptor core conformation.

Our results show that the A338V substitution produces a selective signaling phenotype, with largely preserved Gi protein activation but consistently impaired GRK2 and β-arrestin2 recruitment across complementary experimental configurations. Conformational biosensors further support mutation-dependent changes in both basal and agonist-stabilized receptor conformations, while MD simulations suggest that A338V redistributes the conformational ensemble and membrane interactions of the intrinsically disordered C-terminal tail. Importantly, these molecular and signaling changes are accompanied by enhanced GPR183-mediated migration in differentiated HL-60 cells. Together, our findings support a broader principle whereby a single naturally occurring mutation within an IDR of a GPCR can redistribute its conformational ensemble and selectively alter the recruitment and activation of intracellular protein partners.

## Results

### The A338V Mutation Preserves Agonist Potency, but Reduces Maximal mGsi, GRK2, and β-Arrestin2 Engagement in Direct BRET Assays

To determine whether the A338V substitution (Figure S1) alters proximal receptor signaling, we performed direct intermolecular BRET assays monitoring recruitment of mGsi, GRK2, and β-arrestin2 to overexpressed receptors. WT or A338V GPR183 fused N-terminally to HiBiT and C-terminally to Nluc (HiBiT-GPR183-Nluc) were coexpressed in HEK293A cells with Venus-tagged protein partners: Venus-mGsi, GRK2-Venus or β-arrestin2-Venus. Agonist, 7α,25-dihydroxycholesterol, stimulation induced robust increases in BRET signal in all configurations for the WT receptor, confirming functional receptor activation (Figure 1A–C). In all configurations, these agonist-induced maximal BRET responses for the WT were consistently higher than for the A338V variant (Figure 1A–C with the *F*-test *P* < 0.0001 in the Venus-mGsi (95% CI: 10095–11157 for WT; 6200–7138 for A338V) and β-arrestin2-Venus (95% CI: 6845–7760 for WT; 1816–2260 for A338V) setups and *P* = 0.0026 in the GRK2-Venus setup (95% CI: 3351–4781 for WT; 2086–3229 for A338V)). These results indicate reduced transducer and mGsi engagement in the mutant receptor.

**Figure 1:**
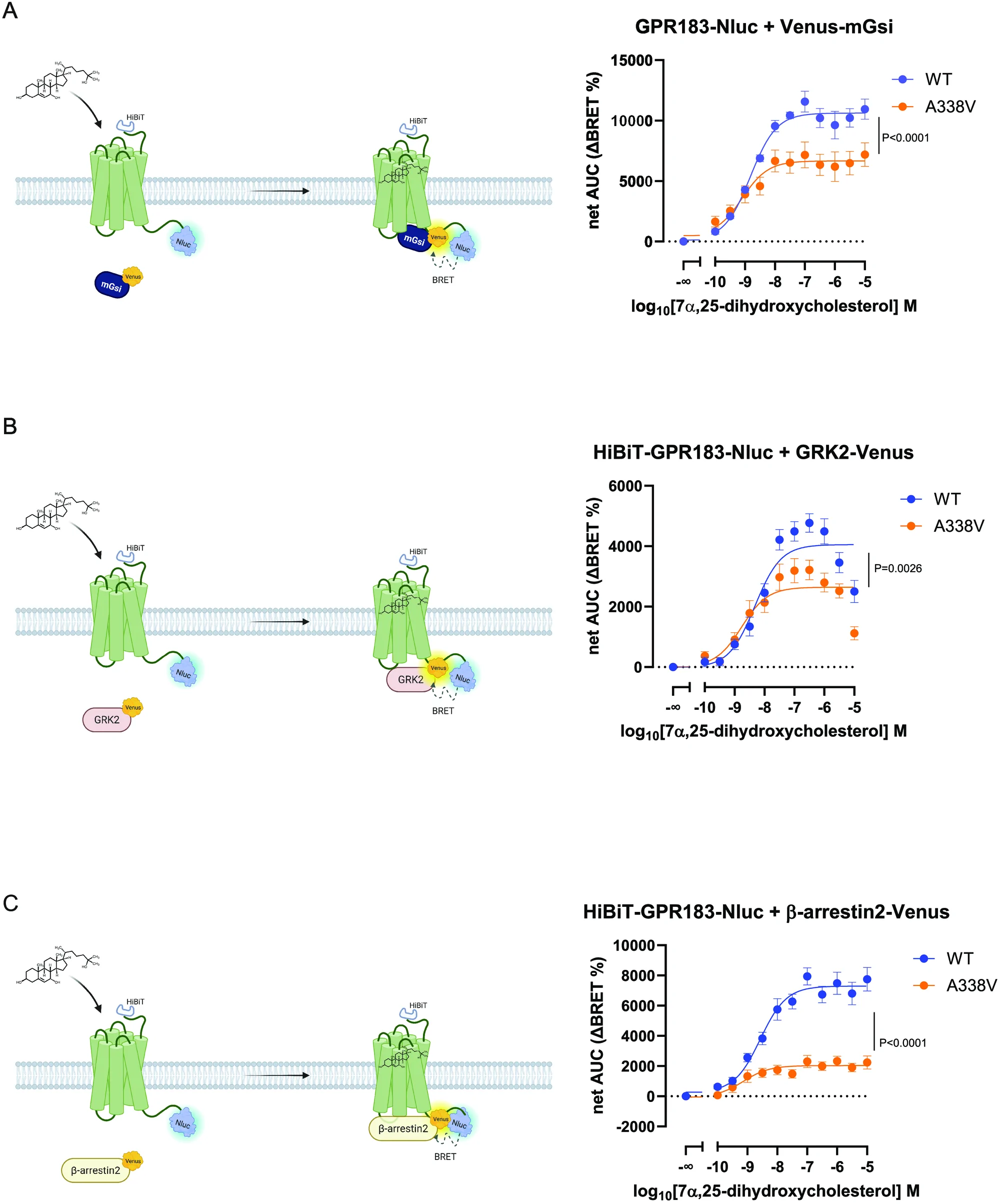
Direct BRET paradigm reveals differences in coupling profiles between GPR183 WT and A338V. (A) Left: Schematic representation of direct BRET-based Venus-mGsi recruitment assay; Right: The concentration response curve of 7α,25-dihydroxycholesterol in this setup. The data are presented as pooled net AUC from ΔBRET % (over three baseline measurements) from 15 min measurements. (B) Left: Schematic representation of direct BRET-based GRK2-Venus recruitment assay; Right: The data are presented as pooled net AUC from ΔBRET % (over three baseline measurements) from 15 min measurements. (C) Left: Schematic representation of direct BRET-based β-arrestin2-Venus recruitment assay; Right: The data are presented as pooled net AUC from ΔBRET % (over three baseline measurements) from 15 min measurements; Net AUC data are presented as pooled from *n* ≥ 3 independent experiments and error bars represent s.d. of net AUC calculations. Please see the Methods section for more details about the calculations. Kinetics data which were used to calculate net AUC are shown in the Figure S2.

Concentration–response curves were fitted to derive *EC*_50_ values, which did not differ significantly between WT and A338V receptors. Thus, agonist potency remained unchanged, indicating that the observed differences reflect altered signaling efficacy and coupling efficiency, rather than impaired ligand recognition. Kinetics plots with data underlying the concentration response curve plots are shown in Figure S2A-C.

Because the A338V substitution resides within the C-terminal tail, 23 amino acids from the distal end—the same region to which Nluc is fused in the two receptor constructs—direct BRET measurements may be sensitive to changes in donor–acceptor proximity or dipole orientation. Reduced BRET efficiency could theoretically arise from altered positioning of the Nluc donor caused by mutation-induced C-tail conformational rearrangements. We therefore sought to validate these findings using assay configurations independent of C-terminal donor geometry.

### Bystander BRET Assays Confirm Impaired GRK2 and β-Arrestin2 Recruitment

To validate the direct BRET findings, membrane-recruitment assays were performed using N-terminally HiBiT-tagged GPR183 WT or A338V, rGFP-CAAX/Venus-KRas (cell membrane markers), and *R*luc2-tagged or Nluc-tagged transducers or effector proteins (Figure 2A–D). In contrast to direct BRET assays, this bystander BRET approach is largely independent of receptor–transducer dipole alignment.

**Figure 2:**
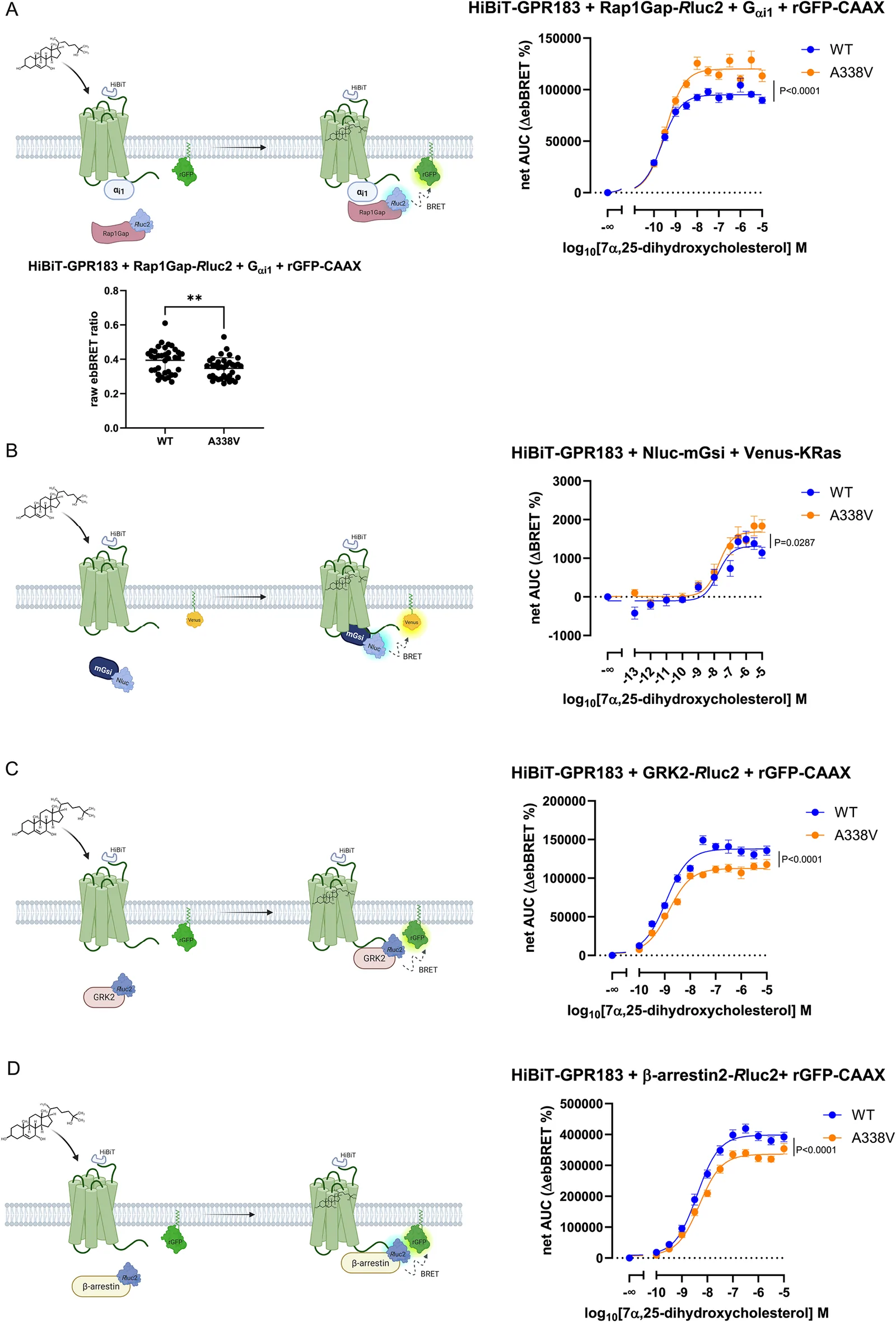
Bystander BRET paradigms demonstrates that GPR183 A338V recruits GRK2 and β-arrestin2 less efficiently than WT. (A) Left: Schematic representation of enhanced bystander BRET (ebBRET) setup to measure Gi activation. Right. The concentration response curve of 7α,25-dihydroxycholesterol in this setup. The data are presented as pooled net AUC from ΔebBRET % (over three baseline measurements) from 15 min measurements. Bottom: Constitutive activity of HiBiT-GPR183 WT and A338V represented as raw ebBRET values prior to ligand stimulation. Each data point represents an individual well. (B) Left: Schematic representation of bystander BRET setup to measure Nluc-mGsi recruitment to the membrane bound receptor. Right. The concentration response curve of 7α,25-dihydroxycholesterol in this setup. The data are presented as pooled net AUC from ΔBRET % (over three baseline measurements) from 15 min measurements. (C) Left: Schematic representation of ebBRET setup to measure GRK2 recruitment. Right. The concentration response curve of 7α,25-dihydroxycholesterol in this setup. The data are presented as pooled net AUC from ΔebBRET % (over three baseline measurements) from 15 min measurements. (D) Left: Schematic representation of enhanced bystander ebBRET setup to measure β-arrestin2 recruitment. Right. The concentration response curve of 7α,25-dihydroxycholesterol in this setup. The data are presented as pooled net AUC from ΔebBRET % (over three baseline measurements) from 15 min measurements. Net AUC data are presented as pooled from *n* ≥ 3 independent experiments and error bars represent s.d. of net AUC calculations. Kinetics data which were used to calculate net AUC are shown in the Figure S3B–E.

In the Rap1Gap-*R*luc2-based Gi activation/translocation assay, the A338V variant exhibited higher agonist-induced ebBRET (enhanced bystander BRET) responses compared to WT (Figure 2A Right-; with the *F*-test *P* < 0.0001; 95% CI: 91601–98603 for WT; 114400–125853 for A338V). However, analysis of basal signaling revealed that A338V displays slightly reduced constitutive Gi activity (*P* = 0.0063; Figure 2A Bottom). Because agonist responses were normalized to basal activity, the lower baseline produced a greater net AUC (area under the curve) for the mutant receptor (Figure 2A Right). When raw agonist-stimulated BRET values were compared directly, WT and A338V responses were largely comparable (Figure S3A), indicating preserved overall Gi activation capacity of the overexpressed cells. Still, similar results were obtained in an independent Gi configuration using Nluc-mGsi and Venus-KRas with the same HiBiT-tagged receptor constructs, where agonist was equally potent but slightly more efficacious on the mutant variant (Figure 2B; the *F*-test *P* = 0.0287; 95% CI: 1049–1647 for WT; 1513–1879 for A338V). In agreement with these Gi-based assays, agonist stimulation of HiBiT-GPR183 A338V-overexpressing HEK293A lead to a statistically significant decrease in forskolin stimulated cAMP levels in comparison with the WT-overexpressing HEK293A cells (Figure S3A; the *F*-test *P* < 0.0001; 95% CI: 7754–9546 for WT; 11824–13400 for A338V).

In contrast, agonist-induced recruitment of GRK2-*R*luc2 and β-arrestin2-*R*luc2 was significantly reduced for A338V relative to WT (Figure 2C,D, with the F-test *P* < 0.0001; 95% CI: 130478–145281 for WT; 107772–117437 for A338V in the GRK2-*R*luc2 setup and 95% CI: 384163–412691 for WT; 322184–350675 for A338V in the β-arrestin2-*R*luc2 setup). Concentration–response analyses revealed similar agonist *EC*_50_ values between receptor, confirming that ligand potency remains unchanged. Kinetics plots underlying the concentration response curve plots are shown in Figure S3B–E.

In agreement with these findings, agonist-induced receptor internalization of the overexpressed HiBiT-GPR183 A338V was significantly impaired in comparison with WT (*P* = 0.0008 for pcDNA vs WT, *P* = 0.0133 for WT vs A338V, *P* < 0.0001 for pcDNA vs A338V; Figure S3F) supporting the findings that A338V could selectively impair GRK2 and β-arrestin2 recruitment.

To determine whether differences in receptor abundance contribute to this phenotype, total and cell surface expression levels of overexpressed WT and A338V HiBiT-GPR183 were assessed in HEK293A cells. Immunoblot analysis revealed stronger total expression of WT compared to A338V (Figure S3G), indicating higher overall abundance of the WT receptor. In contrast, cell surface luminescence measurements demonstrated that the A338V variant exhibits increased plasma membrane expression relative to WT (*P* = 0.0008; Figure S3H). These results indicate that the reduced GRK2 and β-arrestin2 recruitment observed for A338V cannot be attributed to decreased receptor availability at the cell surface. Furthermore, despite higher membrane expression, the A338V variant exhibited slightly reduced basal Gi activity, indicating that the mutation affects constitutive signaling independently of receptor abundance. Overall, these data support a selective effect of the A338V substitution on receptor signaling rather than altered receptor expression.

### Complementary Conformational Biosensors, Including a Newly Developed cpGFP-Based GPR183 Sensor, Reveal Mutation-Dependent Conformational Changes

To determine whether the A338V substitution induces detectable changes in receptor core conformation, we employed our recently published intramolecular conformational biosensor (HiBiT-GPR183-mNG-Nluc, Figure 3A).^22^ Analysis of basal conformational states showed that, at comparable expression levels, basal BRET values were consistently higher for the A338V variant, producing distinct WT and mutant populations (Figure 3B). These data initially suggest that the mutation alters the basal conformational ensemble of the receptor prior to agonist stimulation. However, agonist-induced conformational changes did not differ significantly between WT and A338V receptors (Figures 3C and S4A).

**Figure 3:**
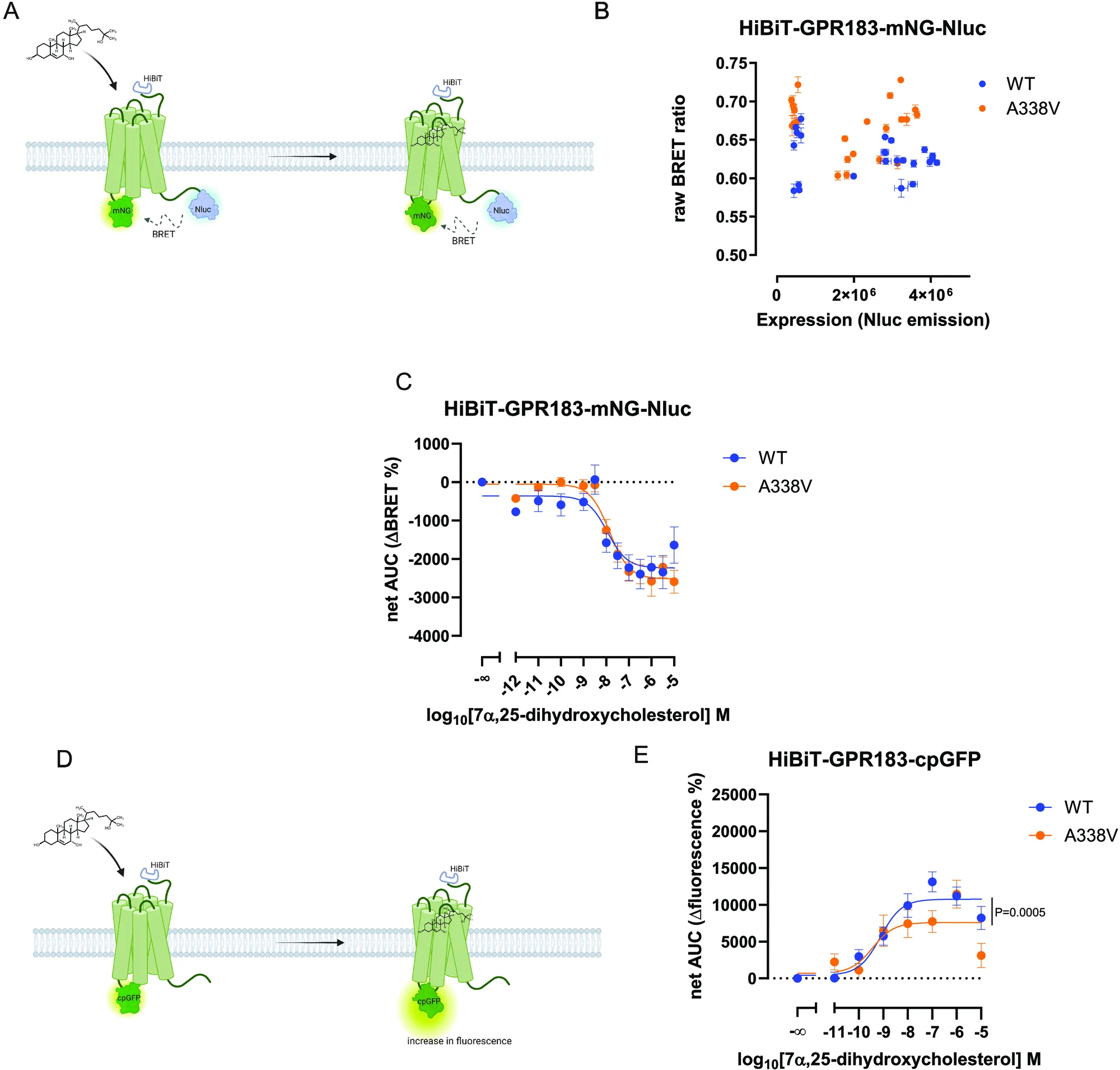
Analysis of conformational dynamics reveals changes in receptor conformation. (A) Schematic representation of the mNG-Nluc BRET-based GPR183 conformational sensor. (B) raw BRET values obtained for the HiBiT-GPR183-mNG-Nluc WT and A338V for different receptor overexpression levels. Each data point indicates one well from three separate plates 0. (C) The concentration response curve of 7α,25-dihydroxycholesterol in this setup. The data are presented as pooled net AUC from ΔBRET % (over three baseline measurements) from 15 min measurements. (D) Schematic representation of the cpGFP-based GPR183 conformational sensor. (E) The concentration response curve of 7α,25-dihydroxycholesterol in this setup. The data are presented as pooled net AUC from Δfluorescence % (over five baseline measurements) from 15 min measurements. Net AUC data are presented as pooled from *n* ≥ 3 independent experiments and error bars represent s.d. of net AUC calculations. Kinetics data which were used to calculate net AUC are shown in the Figure S4A,B.

Nevertheless, because the mNG-Nluc biosensor incorporates Nluc at the C-terminus, the observed BRET differences could reflect both changes in receptor core conformation and mutation-dependent repositioning of the flexible C-tail. To distinguish between these possibilities under agonist-stimulated conditions, we developed circularly permuted GFP (cpGFP)-based conformational biosensors for WT and A338V GPR183 (HiBiT-GPR183-cpGFP), in which cpGFP replaces ICL3 while the C-tail remains untagged (Figure 3D). In this sensor paradigm, agonist-induced outward movement of TM6 is reported as a change in cpGFP fluorescence. Using the cpGFP-based sensors, we found that agonist potency was indistinguishable between WT and A338V receptors (Figures 3E and S4B). In contrast, the maximal fluorescence response was significantly reduced for the A338V variant (F-test, *P* = 0.0005; 95% CI: 9760–11772 for WT; 6204–9005 for A338V), indicating that although both receptors respond to agonist with similar potency, they could adopt distinct agonist-stabilized conformational ensembles. Importantly, these experiments were performed at comparable cell surface expression of the sensors (Figure S4C).

Altogether, the mNG-Nluc biosensor cannot distinguish changes in receptor core conformation from mutation-induced repositioning of the C-tail under basal conditions. Moreover, because the cpGFP sensor is not ratiometric, it cannot reliably report basal conformational differences, as fluorescence intensity may be influenced by well-to-well variation in cell number or sensor expression. Nevertheless, the complementary cpGFP experiments demonstrate that the A338V substitution alters the agonist-induced conformational response of the receptor core despite preserving agonist potency. Together with the altered basal BRET profile and Gi signaling phenotype, these findings suggest that the A338V mutation reshapes both the basal and agonist-stabilized conformational landscapes of GPR183, providing a structural basis for its signaling bias.

### Nluc Dark Experiments Probe C-Terminal Structural Effects

To further investigate the basis of reduced transducer recruitment observed for A338V, we employed an enzyme-deficient Nluc R164Q construct (Nluc* “Nluc dark”; R164Q denotes mutation in the Nluc)^23,24^ and used it fused to the receptor C-tail in the already introduced assays (Figure 4A). In this configuration, the receptor tag does not contribute directly to the measured luminescence signal as luminescence originates from *R*luc2-tagged Rap1Gap, β-arrestin2 and GRK, or Nluc-tagged mGsi and not from the receptor, thereby allowing recruitment to be assessed independently of receptor-derived energy transfer while preserving the structural context of the C-terminal fusion.

**Figure 4:**
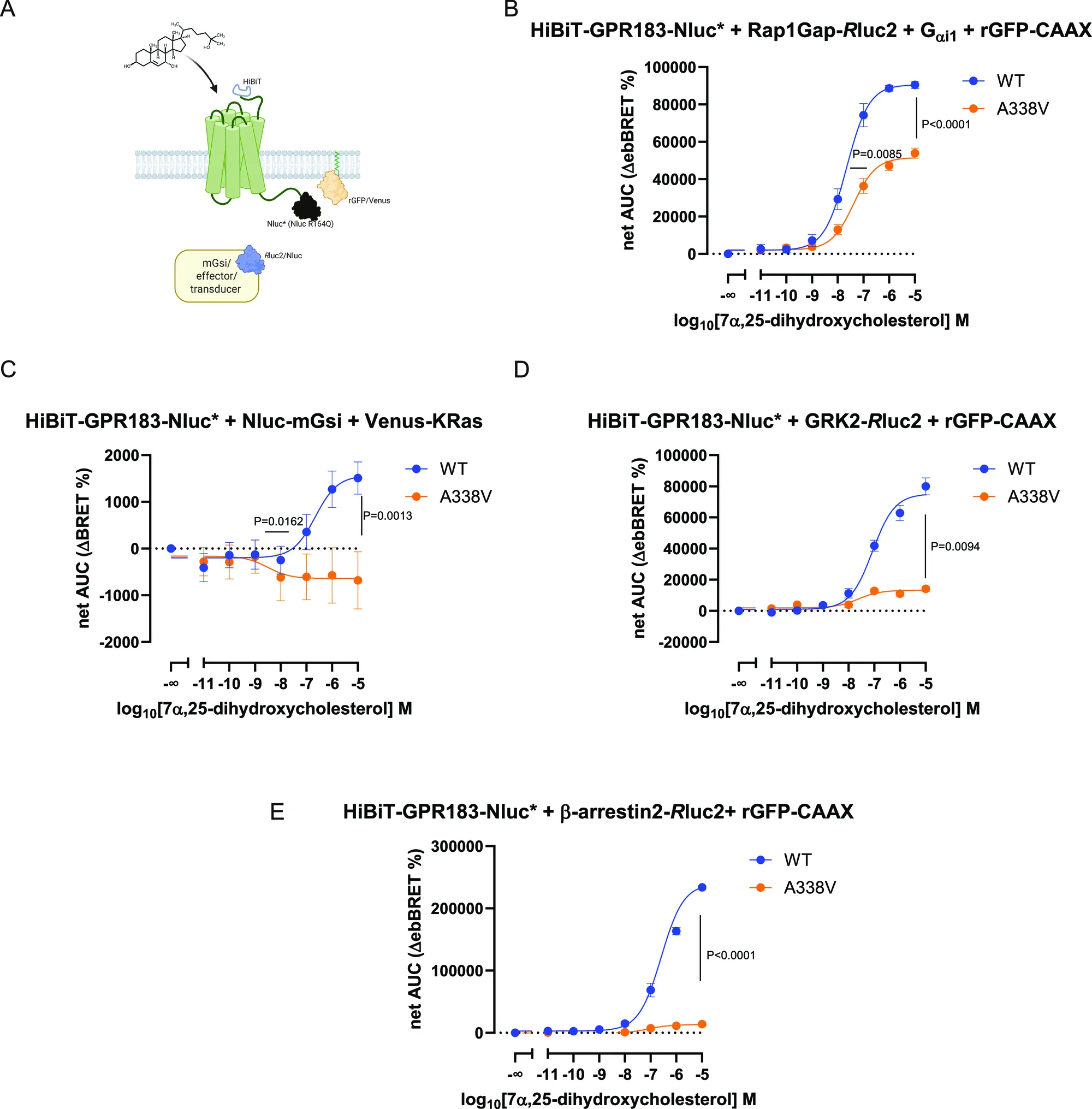
Bystander BRET experiments with C-tail-tagged Nluc* receptors indicate that A338V introduces substantial changes in the C-tail conformation. (A) Left: Schematic representation of different experimental paradigms used with an overexpressed HiBiT-GPR183-Nluc*. (B) The concentration response curve of 7α,25-dihydroxycholesterol in the ebBRET setup to measure Gi activation. The data are presented as pooled net AUC from ΔebBRET % (over three baseline measurements) from 15 min measurements. Right. (C) The concentration response curve of 7α,25-dihydroxycholesterol in the bystander setup to measure Nluc-mGsi recruitment. The data are presented as pooled net AUC from ΔBRET % (over three baseline measurements) from 15 min measurements. (D) The concentration response curve of 7α,25-dihydroxycholesterol in the ebBRET setup to measure GRK2-*R*luc2 recruitment. The data are presented as pooled net AUC from ΔebBRET % (over three baseline measurements) from 15 min measurements. (E) The concentration response curve of 7α,25-dihydroxycholesterol in the ebBRET setup to assess β-arrestin2 recruitment. The data are presented as pooled net AUC from ΔebBRET % (over three baseline measurements) from 15 min measurements. Net AUC data are presented as pooled from *n* ≥ 3 independent experiments and error bars represent s.d. of net AUC calculations. Kinetics data which were used to calculate net AUC are shown in the Figure S5A–D.

Potency of the agonist and the maximal agonist-induced activation of Gi by the membrane-bound HiBiT-GPR183-Nluc* remained significantly reduced for the A338V variant relative to the WT (Figure 4B; *P* = 0.0085 for log* *EC_50_ with 95% CI: −7.72 to −7.59 for WT; −7.62 to −7.15 for A338V, and *P* < 0.0001 for efficacy with 95% CI: 88572–92615 for WT; 47663–55650 for A338V). Interestingly, in the Nluc-mGsi setup, the presence of the Nluc* on the HiBiT-GPR183 A338V C-tail markedly reduced potency and efficacy of the agonist in the recruitment of this conformational probe, and in fact appears to dissociate it from its initial binding at the overexpressed receptor (Figure 4C; *P* = 0.0162 for log EC_50_ with 95% CI: −7.10 to −6.19 for WT; −10.24 to −7.23 for A338V, and *P* = 0.0013 for efficacy with 95% CI: 1227–1955 for WT; −829 to −464 for A338V). Because the Nluc*moiety remains structurally present but does not function as the luminescent donor, this setup allows discrimination between altered BRET efficiency and genuine changes in recruitment.

Persistence of reduced mGsi, GRK2 (Figure 4D; *P* = 0.0094 for efficacy with 95% CI: 66398–84425 for WT; 10045–16844 for A338V), and β-arrestin2 (Figure 4E; *P* < 0.0001 for efficacy with 95% CI: 233401–246352 for WT; 10945–15842 for A338V) engagement in the Nluc* configuration indicates that the observed effects are not solely attributable to donor–acceptor geometry and instead likely reflect mutation-dependent conformational rearrangements within the C-tail which cause steric clashes when the Nluc* is present. Kinetics plots underlying the concentration response curve plots are shown in Figure S5A–D.

Notably, the A338V mutant of the GPR183 fused to Nluc* had significantly lower cell surface expression levels, which could contribute to reduced observed ligand-activated receptor-mediated phenomena, but should not abolish them, as the expression levels of the HiBiT-GPR183-Nluc* A338V construct were still substantial (*P* < 0.0001; Figure S5E). Furthermore, an orthogonal cell-surface expression assay using HiBiT-GPR183-Nluc constructs together with Venus-KRas also revealed significant BRET differences between HiBiT-GPR183-Nluc WT and A338V receptors (*P* = 0.045 with unpaired *t* test, and both significant vs pcDNA with *P* = 0.0007 and 0.0164 for WT and A338V with one-way ANOVA; Figure S5F). However, altered BRET efficiency in this configuration may additionally reflect mutation-dependent changes in donor–acceptor orientation or distance arising from C-tail reorganization. Overall, these results support the interpretation that the A338V substitution alters the conformational organization and spatial accessibility of the C-terminal tail, thereby limiting productive intracellular transducer coupling to an overexpressed GPR183 A338V, particularly when the C-tail is tagged. These suggest that modification of the structural ensemble associated with the tail would be expected to impact the corresponding functionality, as discussed below.

### MD Simulations

#### The Intrinsically Disordered Nature of the GPR183 C-Terminal Tail Favored Deep-Learning–Based Ensemble-Aware Reconstruction Approaches over Conventional Homology Modeling

Comparison of reconstruction strategies indicated that AlphaFold3 produced the most consistent full-length models of GPR183 (Figure 5A), particularly for the unresolved intracellular and C-terminal regions. Relative to AlphaFold2, AlphaFold3 yielded lower RMSD values in comparison to the experimental structures while maintaining favorable SASA and pTM metrics (Figure S6). MODELER-based approaches were less suitable for reconstruction of the highly dynamic C-terminal region because of the extensive absence of homologous structural templates. Consequently, AlphaFold3-derived models were selected as starting points for subsequent molecular dynamics simulations.

**Figure 5:**
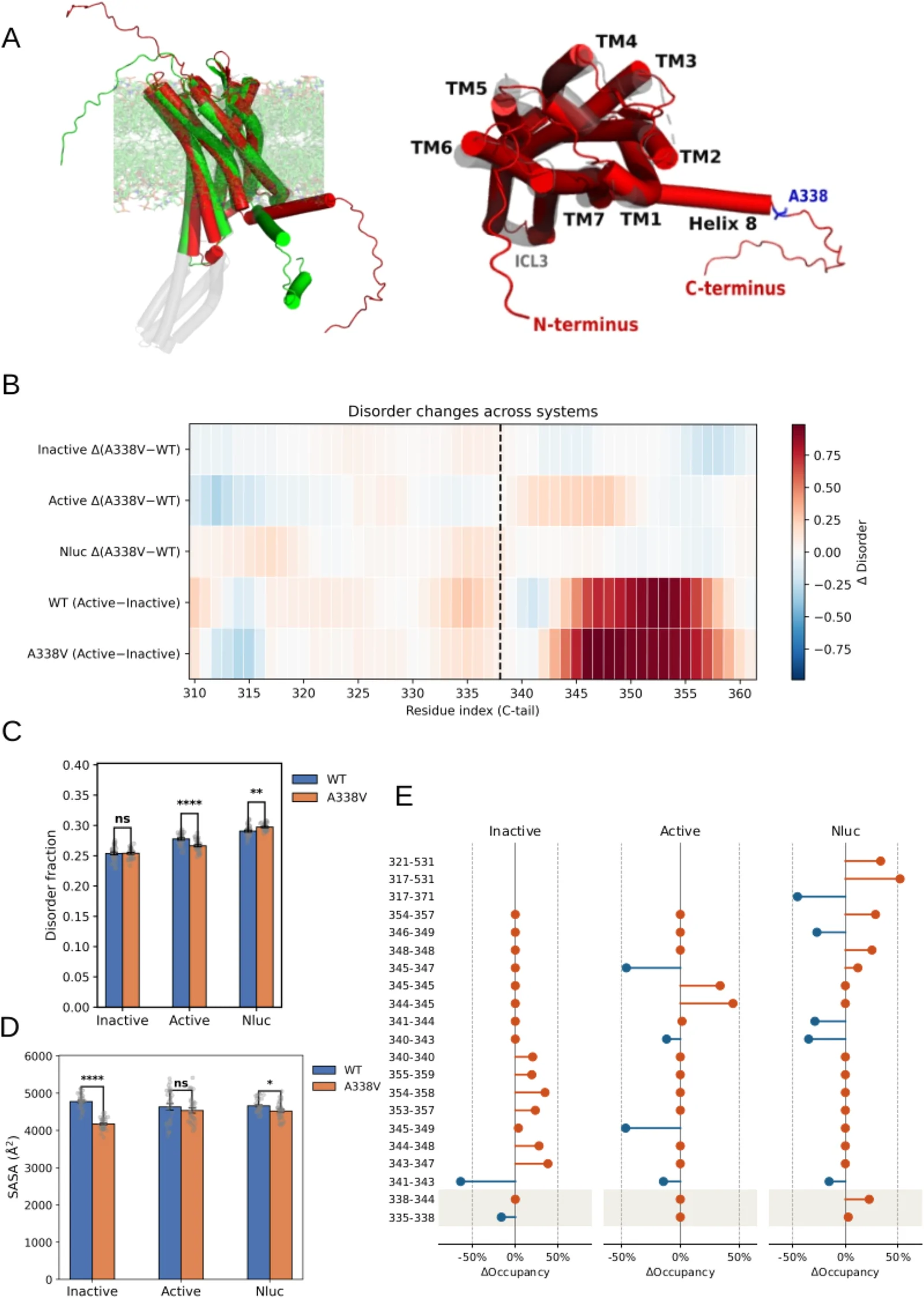
A338V can reshape the C-terminal hydrogen-bonding network, supporting a mutation-driven shift in tail disorder and conformational dynamics. Structural and dynamic effects of the A338V mutation on the GPR183 C-terminal tail. (A) Structural overview of the receptor systems. Left: schematic representation of the experimental cryo-EM structure together with reconstructed inactive (green) and active (red) conformations of GPR183. Right: intracellular view highlighting the transmembrane helices (TM), intracellular loops (ICL), mutated residue 338, and the N- and C-termini. (B) Residue-resolved disorder probabilities for the C-terminal tail derived from DSSP analysis across the simulated systems with residue 338 shown with dashed line. (C) Distribution of the fraction of disordered residues for the full receptor across simulations. (D) Distribution of SASA of the C-terminal tail for all simulated systems. (E) Differences in C-tail hydrogen-bond occupancies upon the A338V mutation across the analyzed systems with H bonds.

#### MD Simulations Reveal Extensive Mutation-Dependent Remodeling of the GPR183 C-Terminal Conformational Ensemble

From the trajectories obtained for each system (the active and inactive structures were used as starting pose for the simulations of GPR183 without the Nluc tag), five representative structural clusters were selected and are shown in the Supporting Information (Figure S7), together with disconnectivity graphs derived from these trajectories (Figures S8–S13) illustrating the topology of the underlying energy landscapes.^25^ Comparison of the WT and mutant systems indicates that the most pronounced differences arise from conformational changes within the C-tail, suggesting that this region plays a central role in mediating the structural effects of the mutation. The distance between the distal and proximal regions of the C-tail varied across systems. No significant alterations in disconnectivity graph topology were observed for the Nluc systems (Figures S8–S9). The most pronounced differences were identified between WT and A338V inactive structures, where the mutant displayed a more compact C-tail conformational topology (Figure S11), consistent with the C-tail DSSP distribution described below.

The C-terminal tail exhibits strong activation-dependent structural changes that are further amplified by the A338V mutation after comparing simulations starting from active and inactive states. DSSP-derived secondary-structure probabilities show that the C-tail of GPR183 is highly dynamic and undergoes pronounced conformational rearrangements upon receptor activation (Figure 5B). In the inactive state, residues 325–335 display relatively low disorder probabilities, suggesting the presence of a transiently structured segment in the proximal portion of the tail. In contrast, the distal C-terminus (residues ∼340–360) becomes strongly disordered in the active receptor, consistent with activation-dependent release of the tail into a flexible conformation. While the A338V mutation has little effect on the disorder profile of the inactive receptor, it increases disorder probabilities in the downstream region of the tail in the active state. This behavior indicates that the mutation enhances activation-induced flexibility rather than locally destabilizing the structure at the mutation site. Interestingly, comparison with the Nluc-fused constructs further shows that the luciferase tag has only a minor effect on the overall disorder profile of the C-tail.

Despite increasing disorder locally in the C-tail, the A338V mutation redistributes conformational flexibility across the receptor. To assess global structural variability, we quantified the fraction of disordered residues across the entire receptor in three functional states: inactive, active, and Nluc-fused (Figure 5C). In the inactive state, the disorder fraction is similar for the WT and the A338V mutant, with no statistically significant differences. In contrast, the active state exhibits a statistically significant reduction in the overall disorder fraction for the A338V variant relative to WT, despite the increased disorder observed locally in the C-tail (Figure 5C). This behavior suggests that the mutation does not uniformly increase structural disorder but instead redistributes conformational flexibility within the receptor.

Consistent with these observations, the A338V mutation promotes a more compact conformational ensemble of the C-terminal tail. Analysis of the SASA shows a general reduction in C-tail exposure in the mutant (Figure 5D), indicating enhanced self-screening of the tail. This effect is most pronounced in the inactive state, suggesting that the mutation stabilizes more compact C-terminal conformations that may influence receptor activation. The effect is less pronounced in the active ensemble, whereas the Nluc-fused system exhibits a statistically significant reduction in C-tail SASA consistent with enhanced compaction.

Hydrogen-bond analysis further demonstrates that A338V reorganizes the hydrogen-bonding network of the C-terminal region (Figure 5E). In both inactive and active ensembles, hydrogen-bonds involving residue 338 decrease in occupancy upon mutation. In the inactive state, the interaction between residues 335–338 decreases by ∼9%, indicating weakened local contacts around the mutation site, while the decrease is smaller in the active ensemble. Beyond the immediate mutation site, however, A338V redistributes hydrogen-bonding throughout the C-terminal segment. Several interactions involving residues 340–360 increase markedly in the mutant, including S343–T347, H354–S358, and I353–S357, whereas native contacts such as Q341–S343, show reduced occupancy. These results indicate redistribution rather than simple loss of hydrogen-bonding interactions.

The mutation also introduces new long-range interactions that alter the spatial positioning of the C-tail. In the inactive ensemble, the mutant forms hydrogen bonds linking the C-terminus with distal regions of the receptor, including contacts involving R317. These interactions are absent or substantially weaker in the WT and may stabilize inactive C-terminal conformations that hinder structural rearrangements associated with activation. Hydrogen-bonds involving the Nluc reporter further reveal that A338V promotes additional contacts between the C-terminal segment and luciferase residues, most prominently with A531, whose occupancy increases by up to ∼ 52%. Several native C-terminal hydrogen bonds are also weakened or lost.

Together, these results suggest that A338V reshapes the structural ensemble of the GPR183 C-terminal tail, promoting increased activation-dependent flexibility together with enhanced compaction and a reorganized hydrogen-bonding network. Such large-scale reorganization of the C-tail may alter interactions with downstream signaling and regulatory partners, providing a structural basis for the functional effects of the mutation.

Because the experimental data indicated selective impairment of GRK2 and β-arrestin recruitment, we next investigated how A338V influences regulatory transducer-associated conformational ensembles. We therefore focused on the intrinsically disordered C-terminal tail (C-tail), which plays a central role in receptor regulation and downstream signaling. This region contains multiple GRK2-targeted phosphorylation sites involved in β-arrestin recruitment.

#### MD Simulations Reveal Mutation-Dependent Reorganization of the GPR183 C-Terminal Regulatory Region and Altered Membrane Interactions of Phosphorylation-Prone Residues

To further investigate the structural basis for the reduced GRK2 and β-arrestin2 recruitment observed for the A338V variant, we performed molecular dynamics (MD) simulations of active GPR183 together with activating 7α,25-dihydroxycholesterol in complex with either G protein or β-arrestin, and analyzed the conformational properties of the intrinsically disordered C-terminal tail (C-tail), a region known to play a central role in GPCR regulation, phosphorylation, and arrestin engagement. The membrane-proximal C-tail contains multiple serine and threonine residues that represent potential GRK2 phosphorylation sites and therefore represents a key structural element coupling receptor activation to downstream regulatory signaling.

In both the G protein– and β-arrestin–associated ensembles, the mutation was predicted to alter the positioning and conformational heterogeneity of the C-tail, indicating mutation-dependent remodeling of the intracellular regulatory interface. Analysis of residue-resolved disorder profiles suggested that the A338V substitution can substantially reorganize conformational flexibility across the C-terminal region (Figure 6A). In the β-arrestin-bound ensemble, residues surrounding the mutation site exhibited a pronounced decrease in disorder relative to WT, particularly within the membrane-proximal segment encompassing residues ∼323–333. A similar, but less pronounced, trend was observed in the G protein–associated ensemble. In contrast, residues located further downstream in the distal C-tail displayed increased disorder in the mutant receptor, indicating redistribution rather than uniform loss of conformational flexibility. These findings indicate that A338V is able to redistribute the structural ensemble of the C-tail rather than simply destabilizing the region.

**Figure 6:**
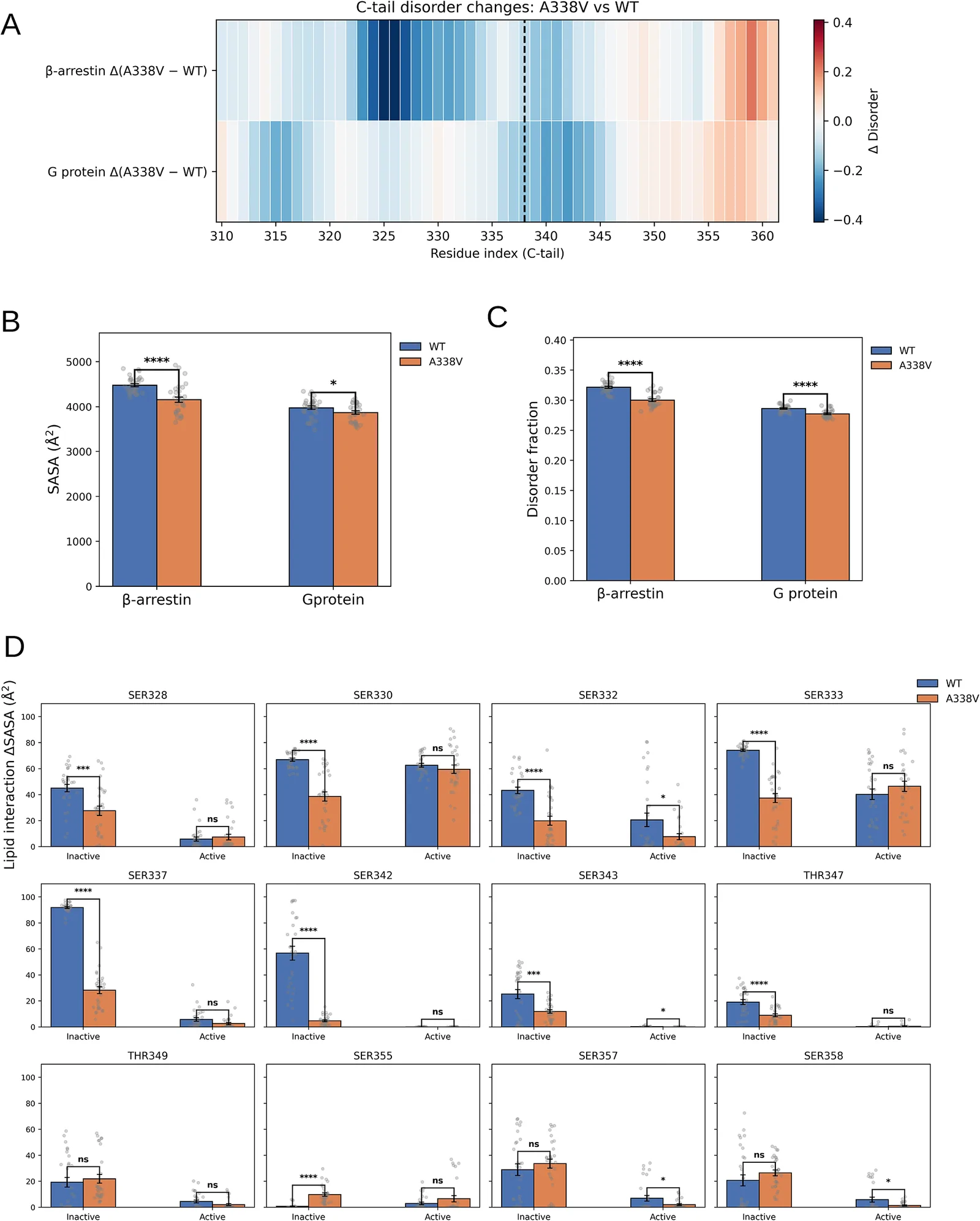
A338V can reorganize the conformational dynamics, compaction, and membrane interactions of the GPR183 C-terminal regulatory tail in G protein– and β-arrestin–associated complexes. (A) Residue-resolved disorder probabilities for the C-terminal tail derived from DSSP analysis across the simulated systems with residue 338 shown with dashed line. (B) Distribution of the fraction of disordered residues for the complexes. (C) Distribution of SASA of the C-terminal tail for simulated systems. (D) ΔSASA analysis reveals mutation-dependent reorganization of lipid interactions at GPR183 C-tail phosphorylation sites.

To quantify the global effects of the mutation on conformational dynamics, we next analyzed the overall disorder fraction of the simulated receptor complexes (Figure 6B). In both β-arrestin– and G protein–associated systems, the A338V mutant displayed significantly lower disorder fractions compared to WT, indicating reduced global conformational flexibility of the intracellular receptor ensemble. The effect was particularly pronounced in simulations containing β-arrestin, consistent with stronger structural restriction of the regulatory C-tail in the mutant receptor. Importantly, these findings align with the experimentally observed reduction in GRK2 and β-arrestin recruitment, and suggest that the mutation promotes conformational states less compatible with productive regulatory transducer engagement.

Because phosphorylation-dependent GPCR regulation strongly depends on accessibility and membrane positioning of C-tail phosphosites, we next quantified the SASA of the C-terminal region (Figure 6C). The A338V mutation significantly reduced C-tail SASA in both β-arrestin– and G protein–associated simulations, indicating enhanced compaction and self-shielding of the intracellular tail. This effect was strongest in the β-arrestin ensemble, where the mutant receptor adopted markedly less exposed conformations relative to WT. Reduced C-tail exposure may provide a structural basis for the impaired GRK2 and β-arrestin recruitment observed experimentally, as phosphorylation-prone residues may become less accessible or improperly oriented for productive kinase and arrestin interactions.

To investigate whether the mutation additionally alters membrane interactions of phosphorylation-prone residues, we performed residue-resolved lipid-contact analysis using ΔSASA-based measurements for serine/threonine residues within the C-tail regulatory region (Figure 6D) of inactive GPR183 and active coupled to G protein. In the WT receptor, the inactive ensemble exhibited strong membrane interactions for several residues, particularly S330, S333, S337, and S342, indicating that the proximal C-tail adopts membrane-associated conformations prior to activation. Transition to the active state was accompanied by substantial reduction of lipid burial for many of these residues, consistent with activation-dependent disengagement of the regulatory tail from the membrane. Such conformational release would be expected to facilitate access of GRK2 to phosphorylation sites and promote downstream β-arrestin recruitment.

The A338V mutation strongly perturbed this membrane interaction network. In the inactive ensemble, the mutant receptor displayed markedly reduced lipid burial at several phosphorylation-prone residues, including S328, S330, S332, S333, S337, S342, and S343. Among these sites, S337 and S342 exhibited the largest reductions relative to WT, suggesting substantial disruption of the membrane-associated organization of the proximal regulatory tail. Despite reduced membrane burial, the mutant receptor did not exhibit enhanced regulatory transducer recruitment experimentally, indicating that increased exposure alone is insufficient for productive signaling. Instead, these findings suggest that A338V disrupts the coordinated structural organization and dynamic presentation of phosphorylation sites required for efficient GRK2 docking and β-arrestin engagement.

The data from these simulations support a model in which the A338V substitution selectively reshapes the conformational and membrane-interaction landscape of the GPR183 C-tail. The mutation could promote a more compact and globally less dynamic intracellular ensemble, while simultaneously disrupting membrane-associated organization of phosphorylation-prone residues. These predicted structural changes provide a plausible mechanistic explanation for the experimentally observed preserved Gi activation and impaired GRK2/β-arrestin recruitment and support a role for the GPR183 C-tail as an allosteric determinant of signaling bias.

#### A338V Redistributes Allosteric Communication toward the C-Terminal Tail and Remodels the Active-State Receptor Core

To further investigate the structural consequences of the A338V mutation, we analyzed allosteric communication networks derived from the MD simulations and could report that the mutation markedly redistributed allosteric communication, with communication pathways shifting from the C-terminal tail in the WT receptor toward TM7 in the A338V variant (Figure 7A). We have also quantified the distance between TM3 and TM6 by monitoring the C^α^ distance between R129^3.50^ and L243^6.34^ (numbers in the superscript indicate residue positions according to the Ballesteros–Weinstein numbering scheme).^26–28^ In both inactive WT and A338V receptors, the overall transmembrane architecture remained highly similar, as reflected by the absence of significant differences in the average TM3–TM6 distance (Figure 7B,C). In contrast, the active-state A338V receptor exhibited a significant increase in the R129^3.50^–L243^6.34^ distance relative to WT, indicating selective remodeling of the active-state transmembrane core and a redistribution of the active conformational ensemble (Figure 7B,C). Comparison of the Nluc-tagged systems further revealed enhanced allosteric communication within the C-terminal region of the A338V receptor relative to WT (Figure 7D), providing a structural explanation for the increased sensitivity of the mutant to C-terminal modification observed experimentally. In the G protein-bound complexes, the overall allosteric communication was less pronounced and differences between WT and A338V were comparatively subtle, likely reflecting stabilization of the intracellular receptor interface upon G protein binding (Figure 7E). In contrast, β-arrestin-bound complexes displayed substantial redistribution of allosteric communication throughout the receptor core in the A338V variant compared with WT (Figure 7F), consistent with the reduced β-arrestin recruitment observed experimentally. Collectively, these findings suggest that the A338V mutation rewires long-range intramolecular communication while largely preserving the inactive-state receptor core architecture. In the active state, however, the increased TM3–TM6 separation together with the redistribution of allosteric communication indicates selective remodeling of the receptor core, providing a structural hypothesis for the altered signaling and transducer coupling observed experimentally.

**Figure 7:**
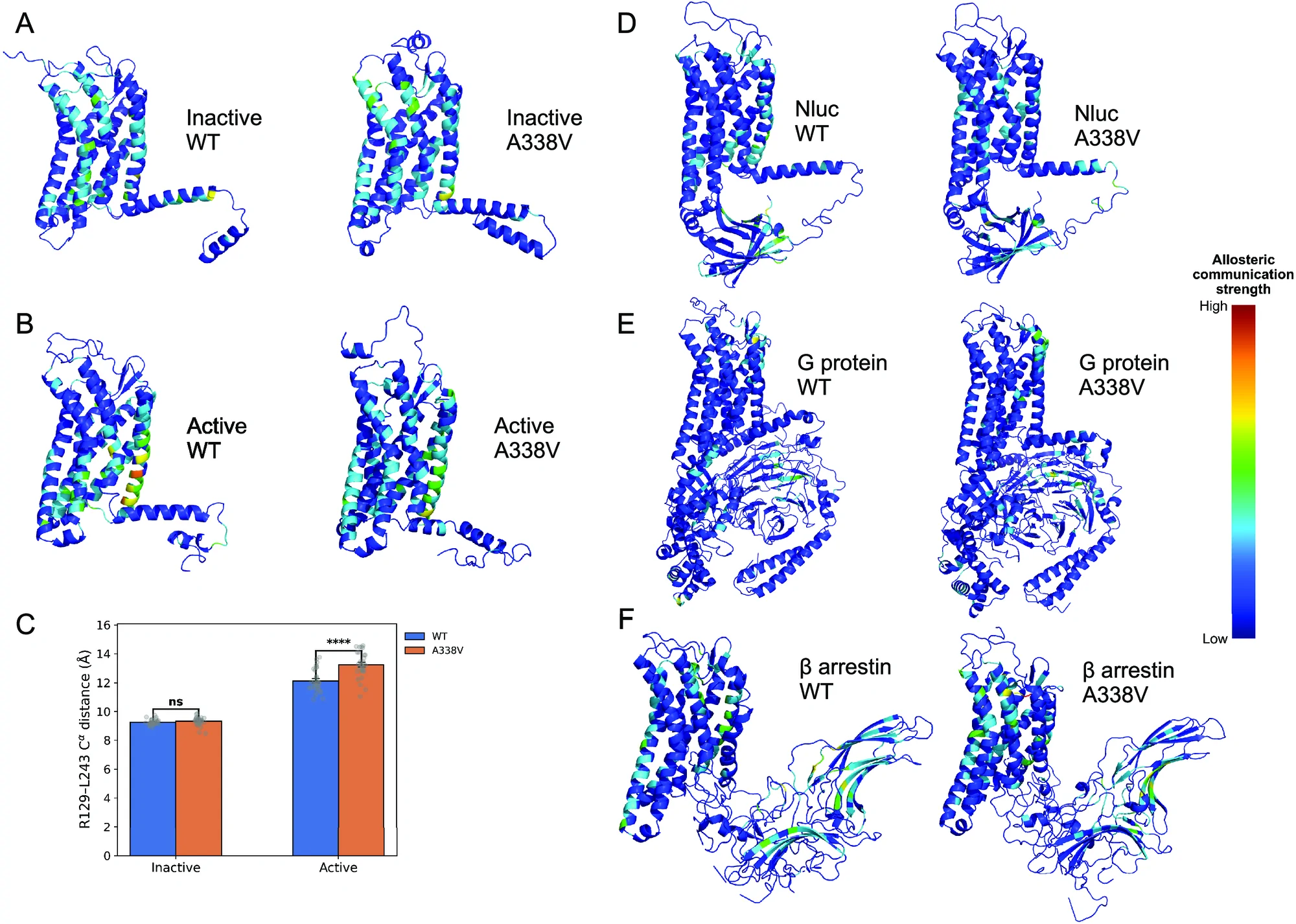
A338V can rewire allosteric communication in a state-dependent manner while selectively remodeling the active-state receptor core. (A) Allosteric communication networks of the inactive WT and A338V receptors reveal redistribution of long-range communication from the C-terminal tail toward TM7 in the mutant. (B) Allosteric communication networks of the active WT and A338V receptors. (C) Quantification of the TM3–TM6 separation, measured as the C^α^ distance between R129^3.50^ and L243^6.34^, shows no significant difference between WT and A338V in the inactive state, whereas the active-state A338V receptor exhibits a significantly increased TM3–TM6 distance relative to WT. (D) Comparison of Nluc-tagged receptor simulations demonstrates enhanced allosteric communication within the C-terminal region of the A338V receptor relative to WT. (E) G protein-bound receptor complexes exhibit generally weaker allosteric communication, with only subtle mutation-induced differences. (F) In β-arrestin-bound complexes, the A338V mutation markedly redistributes allosteric communication throughout the receptor core, consistent with the reduced β-arrestin recruitment observed experimentally.

#### A338V Enhances Migration of Neutrophil-like Cells to the Chemoattractant

To determine whether the effects of the A338V variant on proximal signaling and C-tail conformational dynamics translate into altered receptor function in a more global cellular context, we performed cell migration assays. GPR183 is best known for its role in directing the migration of immune cells in response to oxysterol gradients. We used HL-60 cells, which typify AML, and we differentiated them into neutrophil-like cells to enhance their migratory capacity before electroporation with either WT or A338V HiBiT-GPR183. Next, the cells were allowed to migrate through 5 μm transwell membranes toward 10 nM 7α,25-dihydroxycholesterol for 3 h. Despite comparable cell surface expression of WT and A338V receptors (Figure 8A), overexpression of the A338V variant resulted in a reproducible and significant increase in the proportion of migrated cells compared with the WT (*P* = 0.0451; Figure 8B), demonstrating that the A338V mutation enhances GPR183-mediated chemotactic responses. Together, these results establish a functional link between the altered proximal signaling and structural impact of the A338V mutant and enhanced cellular migration. Although the present study does not address disease mechanisms directly, the association of A338V with AML suggests that these altered migratory properties may be relevant in a pathological context.

**Figure 8:**
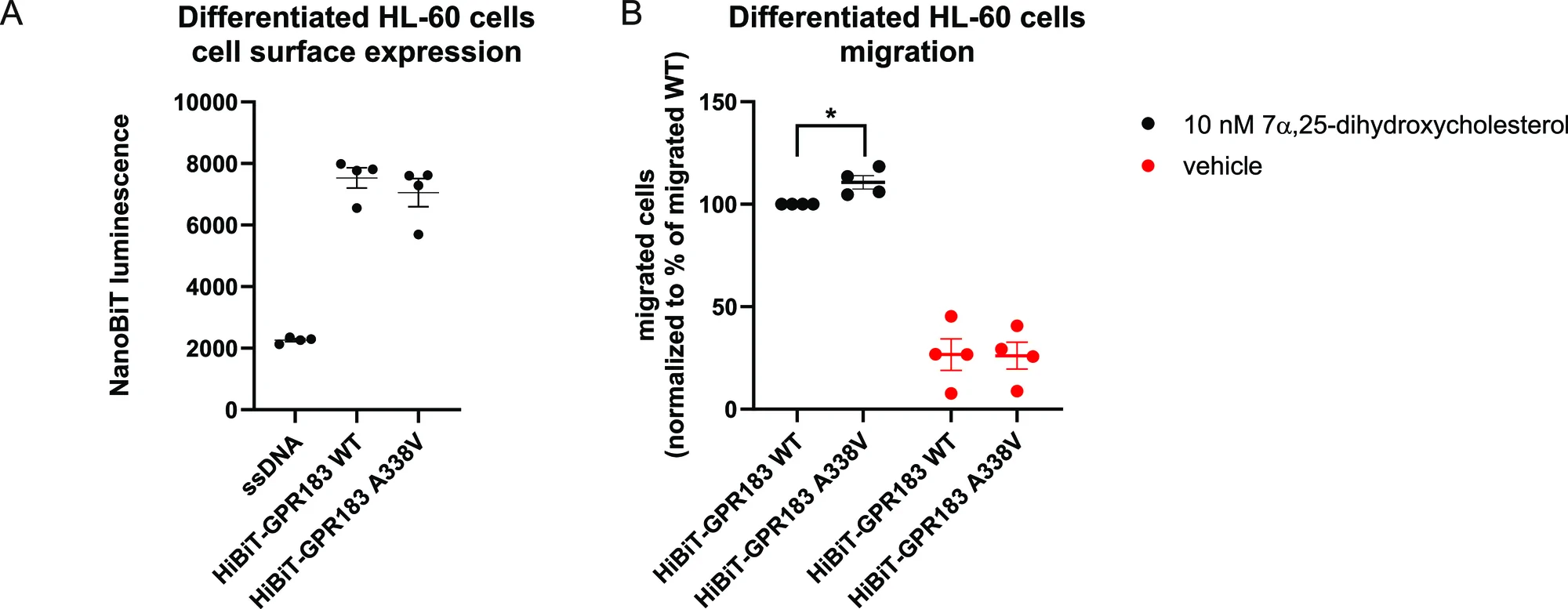
A338V enhances migration of differentiated HL-60 toward chemoattractant. (A) Cell surface expression of HiBiT-GPR183 WT or A338V in differentiated HL-60 cells. The data are represented as mean ± s.e.m. of *n* = 4 independent experiments; ssDNA-transfected cells were used as a control. (B) Analysis of migration through 5 μm pores toward 10 nM 7α,25-dihydroxycholesterol of receptor-overexpressing differentiated HL-60. The data are normalized to the HiBiT-GPR183 WT condition with the chemoattractant and shown as mean ± s.e.m. from *n* = 4 independent experiments. Differences in the migration to 7α,25-dihydroxycholesterol between the WT and A338V were analyzed with one-sample *t*-test.

## Discussion

The present study shows that a single naturally occurring mutation within the intrinsically disordered C-terminal tail of GPR183 can selectively reshape receptor signaling without globally impairing receptor function. Across complementary experimental configurations, A338V largely preserves Gi protein activation while consistently reducing GRK2 and β-arrestin2 recruitment, and these molecular changes are accompanied by enhanced GPR183-mediated migration. The molecular dynamics simulations provide structural insight into how mutation-dependent changes in the C-terminal conformational ensemble may contribute to this selective signaling phenotype. A key experimental result is that the A338V mutation preserves agonist potency and overall Gi signaling capacity, as evidenced by both direct and bystander BRET assays. Although basal Gi activity was modestly reduced, agonist-stimulated Gi activation remained largely comparable or even higher than the WT receptor across multiple assay configurations. Similarly, maximal decrease in cAMP levels was larger in the agonist-stimulated HiBiT-GPR183 A338V-overexpressing HEK293A cells than in the WT-overexpressing counterparts. These observations indicate that the mutation does not impair ligand recognition or the ability of the receptor core to adopt an active conformation capable of engaging G proteins. Consistent with these observations, two complementary intramolecular conformational biosensors revealed mutation-dependent changes in GPR183 conformation. The mNG-Nluc BRET sensor detected differences in the global basal conformational state between WT and A338V receptors. Because Nluc is located at the C-terminus, this signal likely reflects a combination of changes in the transmembrane core and the conformational ensemble of the intrinsically disordered C-tail. Although this BRET-based biosensor cannot directly resolve these individual contributions, an altered basal conformational landscape provides a plausible explanation for the reduced constitutive Gi activity of the A338V variant observed in the ebBRET assays.

In contrast, to provide an independent conformational readout while preserving the unmodified C-terminal tail, we developed a cpGFP-based conformational biosensor for GPR183. The cpGFP biosensor, which reports receptor core rearrangements independently of any C-tail fusion, demonstrated identical agonist potency but a significantly reduced maximal fluorescence response for A338V. These findings indicate that the mutant receptor adopts a distinct agonist-stabilized conformational ensemble. These findings indicate that the mutant receptor adopts a distinct agonist-stabilized conformational ensemble. Together, these complementary measurements support the conclusion that the A338V substitution reshapes both the basal and agonist-bound conformational landscapes of GPR183, providing a structural basis for its altered signaling profile. Next, the other set of wet-lab experiments consistently revealed a marked reduction in GRK2 and β-arrestin2 recruitment for the A338V variant. This effect was observed across both direct and bystander BRET assays as well as in the internalization assays following agonist stimulation in which the A338V variant was slightly less internalized. Together, these orthogonal approaches strongly indicate that the observed reduction in transducer engagement reflects a genuine functional consequence of the mutation rather than a technical artifact. Importantly, this impairment occurred despite increased plasma membrane expression of the mutant HiBiT-GPR183 receptor construct (without the C-tail fusion) in overexpressed HEK293A cells, excluding reduced receptor availability as an explanation and instead pointing to altered receptor–transducer coupling efficiency. Along these lines, GRK2 and β-arrestin2 interact largely with the GPCRs C-tail,^12,29^ so that a different, less accessible conformation of this domain in GPR183 A338V may explain reduced engagement of both proteins. Additionally, experiments with Nluc-dark R164Q confirmed that A338V is less accessible to transducer proteins.

Functionally, the receptor remains equally competent for Gi activation but is substantially more deficient in the recruitment of GRK2 and β-arrestin2, indicating that the mutation introduces pathway bias. This selective signaling profile may have implications in disease contexts such as AML, where altered receptor regulation, rather than complete loss or gain of function, could influence cellular behavior. Consistent with this possibility, overexpression of HiBiT-GPR183 A338V in HL-60 cells, a human AML cell line differentiated into neutrophil-like cells, significantly enhanced migration toward the endogenous agonist 7α,25-dihydroxycholesterol. Previous studies have shown that GPR183-directed cell migration is coordinated by both Gi activation and β-arrestin recruitment.^6^ Our findings therefore suggest that the A338V mutation enhanced migratory responses despite impaired β-arrestin2 engagement. Although the present study does not aim to address a role for GPR183 A338V in AML, these observations raise the possibility that signaling bias arising from mutations within the intrinsically disordered C-tail may contribute to disease-associated cellular phenotypes.

The MD simulations provide a structural framework for interpreting these experimental observations. The A338V substitution induces widespread reorganization of the C-terminal tail, including increased activation-dependent disorder, reduced solvent exposure indicative of tail compaction, and a redistribution of hydrogen-bonding interactions. Notably, the mutation promotes the formation of alternative intratail and long-range contacts, including interactions linking the C-terminus to other intracellular regions. These changes suggest that the mutation reshapes the conformational ensemble and spatial positioning of the C-tail, rather than causing a simple local perturbation. The results further indicate that A338V modifies the activation-dependent dynamics of the C-terminal region. In the WT receptor, activation is associated with increased disorder of the distal C-terminal tail, consistent with release of this region into a more flexible conformational state. This behavior is in line with previous studies showing that IDRs often become more prominent during GPCR activation, facilitating conformational adaptability and signaling interactions.^18^ The mutation amplifies this behavior by increasing disorder in the downstream portion of the tail while simultaneously promoting compaction of the C-terminal ensemble. Such a redistribution of structural flexibility suggests that the mutation alters the dynamic coupling between the receptor core and the regulatory C-terminal region.

Importantly, the structural changes observed in silico align closely with the functional phenotype measured in cells. The C-terminal tail of GPCRs is a critical platform for GRK phosphorylation and β-arrestin binding. The increased compaction and altered hydrogen-bonding network observed for A338V may reduce accessibility or alter the presentation of interaction motifs required for efficient recruitment of regulatory transducers. At the same time, the preservation of global receptor activation and Gi coupling is consistent with the comparatively limited structural changes observed within the transmembrane core in the simulations and with the conformational biosensor data. The allosteric communication analysis further extends this structural interpretation by demonstrating that the A338V mutation rewires long-range intramolecular communication in a state-dependent manner. Although the inactive WT and A338V receptors displayed comparable transmembrane core architectures, the mutation redistributed communication pathways from the C-terminal region toward TM7, indicating that subtle changes in communication can occur in the absence of major structural rearrangements. In contrast, the active-state receptor exhibited both redistribution of allosteric communication and an increased TM3–TM6 separation, suggesting selective remodeling of the active conformational ensemble. Moreover, enhanced communication within the C-terminal region of the Nluc-tagged A338V receptor provides a structural explanation for its increased sensitivity to C-terminal modification, whereas the pronounced redistribution of communication observed in the β-arrestin-bound complex is consistent with the reduced β-arrestin recruitment measured experimentally. Together, these findings indicate that the A338V mutation primarily acts by rewiring the transmission of allosteric information rather than by globally destabilizing the receptor structure.

The Nluc-based simulations further suggest that the mutation may influence the spatial relationship between the C-tail and fused reporter domains, reflecting altered tail positioning. However, the persistence of reduced recruitment in configurations independent of receptor-derived luminescence argues that steric or conformational constraints imposed by the mutated tail itself are the primary driver of the observed phenotype, rather than tag-related artifacts.

More generally, our findings support the concept that IDRs of GPCRs can encode selective transducer coupling through their conformational ensembles, such that even a single-residue substitution at a receptor distant end can alter intracellular protein engagement without globally disrupting receptor activation. The simulations also suggest that the mutation can influence the spatial positioning of the C-terminal region relative to the reporter domain in the Nluc constructs. The formation of additional hydrogen-bonds between the C-tail and luciferase residues in the mutant indicates increased proximity and structural coupling between these regions. These structural changes provide a plausible explanation for the altered behavior observed in the BRET-based experiments, as changes in the conformational ensemble of the C-tail would be expected to influence the relative orientation and distance between the receptor and the reporter domain.

Overall, our results indicate that the A338V mutation primarily causes reshaping the structural ensemble of the GPR183 C-terminal tail, rather than inducing a simple local destabilization at the mutation site. Because the C-terminal region of GPCRs often mediates interactions with signaling and regulatory partners, such changes in conformational organization may influence receptor coupling and downstream signaling. The observed mutation-induced redistribution of flexibility, allosteric communication, and hydrogen bonding interactions therefore provides a mechanistic framework for understanding how a single residue substitution in the C-tail can propagate structural effects across the receptor and modulate its functional behavior.

The hypothesis that highly dynamical protein sequences are intrinsically multifunctional has been associated with multifunnel energy landscapes in previous work.^20,21,25^ This functionality would presumably require evolution of a delicately balanced structural ensemble, supporting a variety of motifs that would be stabilized by binding of a particular partner. Mutations that disturb the equilibrium occupation of different motifs would then affect the functionality associated with the associated binding. The present results provide insight at an atomistic level into how the equilibrium is perturbed by mutation and suggest a specific mechanism for the mutational impact on function in terms of the changes in both structure and dynamics.

A particularly interesting observation emerging from the simulations is that the A338V mutation alters, not only the intrinsic conformational properties of the C-terminal tail, but also its interactions with the membrane environment and the presentation of phosphorylation-prone residues. In the WT receptor, several serine and threonine residues located within the proximal C-tail exhibited substantial membrane interactions in the inactive ensemble, whereas receptor activation promoted partial release of this region from the membrane. Such activation-dependent disengagement may facilitate GRK access to phosphorylation sites and thereby support productive β-arrestin recruitment. By contrast, the A338V mutant displayed a markedly reorganized membrane-interaction pattern, with reduced and redistributed lipid burial across multiple phosphorylation-prone residues. Importantly, this altered membrane association was accompanied by increased compaction and reduced global disorder of the intracellular receptor ensemble. Together, these observations suggest that efficient regulatory transducer recruitment depends not simply on phosphosite exposure, but on the dynamic spatial organization of the C-tail relative to both the membrane and receptor core. In this context, the A338V mutation appears to uncouple activation-dependent tail rearrangement from productive downstream regulatory engagement, thereby providing a mechanistic explanation for the preserved Gi activation but impaired GRK2 and β-arrestin2 recruitment observed experimentally. Thus, the A338V variant demonstrates how sequence variation within an IDR of a GPCR can reshape selective intracellular coupling and translate into altered cellular behavior.

## Supplementary Material



## Data Availability

All *in vitro* data sets generated and analyzed during the current study are available from the corresponding author upon reasonable request. Molecular dynamics (MD) simulation trajectories have been deposited in ZENODO and are publicly available under the following repositories: *GPR183 Active, Inactive, and Nluc Molecular Dynamics Trajectories* (*Part 1*) (DOI: 10.5281/zenodo.20268059), *GPR183 Active, Inactive, and Nluc Molecular Dynamics Trajectories* (*Part 2*) (DOI: 10.5281/zenodo.20271359), and *WT/A338V GPR183 Molecular Dynamics Simulations with G Protein and* β*-Arrestin* (DOI: 10.5281/zenodo.20276394). These repositories include all independent simulation replicas used for the analyses presented in this study.

## Notes

Additional experimental and computational details and methods for GPR183 structural model reconstruction, molecular dynamics simulations and energy landscape analysis, allosteric communication analysis, cell-based signaling assays, conformational biosensor experiments, cAMP measurements, receptor internalization, cell migration and immunoblotting; GPR183 A338V mutation schematic, BRET and FRET signaling assays, receptor conformational biosensors, receptor expression and internalization controls, AlphaFold2/3 model validation, conformational cluster analysis, and disconnectivity graphs showing mutation-induced redistribution of the GPR183 C-terminal conformational ensemble (Figures S1–S13) (PDF)
