# Supplementary material for: The AML-Associated A338V Mutation Rewires GPR183 Signaling through C‑Terminal Ensemble Redistribution
Source: J Am Chem Soc. 2026 Oct 8;148(41):44228–44. doi: 10.1021/jacs.6c10287 (PMC13693396; doi:10.1021/jacs.6c10287)
Supplement: Supplementary file 1 [file ja6c10287_si_001.pdf]

# Supporting Information

# The AML-Associated A338V Mutation Rewires GPR183 Signaling Through C-Terminal Ensemble Redistribution

*Louise Andersson [1#], Patryk A. Wesołowski [1,2#], Laura Jahrstorfer [1], Alexander De Rosa [1], Tomáš Heger [2], Vilmos Neuman [3], Adam K. Sieradzan [4,5], David J. Wales [2], Paweł Kozielewicz [1*]*

Authors' addresses

[1] Molecular Pharmacology of GPCRs, Department of Physiology and Pharmacology, Karolinska Institutet, 171 65 Solna, Stockholm, Sweden

[2] Yusuf Hamied Department of Chemistry, University of Cambridge, Lensfield Road, Cambridge, CB2 1EW, United Kingdom

[3] Department of Chemistry, Physical and Theoretical Chemistry Laboratory, University of Oxford, South Parks Road, Oxford OX1 3QZ, United Kingdom

[4] Department of Theoretical Chemistry, University of Gdansk, Wita Stwosza 63, 80-308 Gdansk , Poland

[5] Samsung Medical Center, Sungkyunkwan University School of Medicine, 81, Irwon-ro, Gangnam-gu, Seoul 06351, Korea

[#] - equal contribution

[*] - corresponding author, e-mail: pawel.kozielewicz@ki.se

## Material and methods

*Structural Preparation and Model Reconstruction*

Cryo-EM structures of human GPR183[1] contain substantial unresolved regions that preclude direct use in molecular simulations. The inactive structure (PDB ID: 7TUY) lacks information for residues 1-23, 94-99, 175-179, 187-192, 226-235, 275-280, and 321-361 (C-tail), while the active structure (PDB ID: 7TUZ) lacks residues 1-21, 94-99, 174-178, 317-361 (C-tail). These missing segments include intracellular loops and large portions of the C-terminal region. In particular, the leukemia-associated A338V mutation is located within the unresolved C-terminus, necessitating full-length model reconstruction. The inactive structure (7TUY) contains a BRIL insertion replacing ICL3, along with anti-BRIL and anti-Fab nanobody domains used for cryo-EM stabilization. All non-native domains were removed, the native ICL3 sequence restored, and the bound inverse agonist (PDB ID: KKF) deleted. For the active structure (7TUZ), the heterotrimeric. G protein and activating 7α,25-dihydroxycholesterol ligand were initially removed to ensure consistency across simulations.

*Loop Reconstruction and Mutation Modelling*

The experimental structures used for active and inactive states contained sequence differences (e.g., replaced sequence in ICL3, residues 226-235), resulting in different sequences being used for AlphaFold3[2] input. The resulting structures were subsequently unified and refined using Modeller's functionality ModLoop.[3] The rest of the missing residues were reconstructed using comparative modelling approaches based on AlphaFold2-Multimer, AlphaFold3, and MODELLER/ModLoop. Final models from AlphaFold2-Multimer,[4] AlphaFold3,[2] and MODELLER [3, 5] were selected according to stereochemical quality, compatibility with resolved

cryo-EM coordinates, and structural metrics including RMSD with respect to the original experimental structure, solvent-accessible surface area (SASA), and predicted template modelling (pTM/ipTM) scores. Overall, AlphaFold3 provided the most consistent reconstruction quality and was therefore used for subsequent simulations, while ModLoop was additionally applied for local refinement of intracellular loop regions. The A338V substitution was introduced into both active and inactive conformations followed by local refinement. For the luciferase-attached model, we generated structures using AlphaFold3 with the full amino-acid sequence of GPR183 fused to Nluc and used the resulting model for subsequent simulations.

Six systems were initially constructed: GPR183 WT inactive, GPR183 WT active, GPR183-Nluc WT, GPR183 A338V inactive, GPR183 A338V active, and GPR183-Nluc A338V. Additionally, based on the active receptor structure, four further systems were prepared to investigate the influence of the A338V mutation in the context of downstream signaling complexes. In these systems, the agonist and either heterotrimeric G protein or β-arrestin were retained, generating WT and A338V complexes for both signaling partners. 7α,25-dihydroxycholesterol was parametrized using gaff2 [6] and its position was taken from the position of activating ligand in the 7TUZ reference structure. In the β-arrestin–bound complexes, residues SER328, SER330, SER332, SER333, SER337, SER342, SER343, SER355, SER357, SER358, and THR347 and THR349 were prepared in their phosphorylated states for the MD simulations. Together, this setup yielded four additional systems: GPR183 WT–G protein, GPR183 A338V–G protein, GPR183 WT–β-arrestin, and GPR183 A338V–β-arrestin.

*Molecular dynamics*

Each system was embedded in a POPC bilayer using the Packmol–Memgen protocol,[7] solvated with explicit TIP3P water[8] and neutralized to 0.15 M NaCl. A minimum 10 Å buffer was maintained between the protein and periodic box boundaries. Proteins were described with the AMBER ff14SB force field,[9] lipids with Lipid17,[10] and water with the TIP3P model.[8] Molecular dynamics simulations followed previously established protocols.[11] Each system underwent two-stage energy minimization: (i) 1000 steps (400 steepest-descent, 600 conjugate gradient) with harmonic restraints ($k = 100$ kcal mol$^{-1}$ Å$^{-2}$) applied to all solute atoms, followed by (ii) 2000 steps (800 steepest-descent, 1200 conjugate gradient) with identical restraints applied to heavy atoms only. Equilibration was performed in three stages: (i) heating from 10 to 300 K over 10 000 steps (1 fs, NVT ensemble) with restraints on heavy atoms; (ii) 100 000 steps (1 fs, NPT ensemble) to stabilize density, with heavy-atom restraints maintained; and (iii) 400 000 steps (2 fs, NPT ensemble) with restraints applied to $C^{\alpha}$ and $C^{\beta}$ atoms. Production simulations were conducted in the NVT ensemble at 300 K using a Langevin thermostat, without restraints. Three independent trajectories of $100 \times 10^6$ steps (2 fs timestep) were generated for each system, corresponding to 600 ns total sampling per system. In all explicit solvent simulations, the Particle Mesh Ewald method with a cutoff for nonbonded interactions set at 9 Å was used to speed up calculations.

*Molecular dynamics analysis*

Trajectory analysis was performed to characterize residue interactions, conformational heterogeneity, and the underlying energy landscape. Conformational clustering was carried out using the agglomerative hierarchical clustering algorithm as implemented in cpptraj,[12] yielding representative structures corresponding to the most highly populated states. Secondary structure evolution along the trajectories was analyzed using the DSSP algorithm in cpptraj.[12]

Energy landscape organization was examined using the MDDG framework[13] in combination with disconnectivity graph analysis.[14] MDDG provides a coarse-grained representation of the sampled energy landscape by constructing disconnectivity graphs from the potential energy time series. Proxy minima and transition states were identified via Savitzky–Golay smoothing of the energy signal.[15] In disconnectivity graphs, energy increases along the vertical axis, while branch termini correspond to local minima. Minima are arranged horizontally to reflect funnel organization, with superbasins of minima defined by connectivity below a specified energy threshold.[14]

Solvent-accessible surface area (SASA) was computed to quantify global conformational changes using cpptraj, alongside hydrogen-bond analysis. For each system, the SASA of the C-tail of GPR183 was calculated from three independent 200 ns simulations, yielding a total of 6000 frames. Statistical uncertainties were estimated via block averaging with a block size of 200 frames, exceeding the correlation time of the C-tail SASA, ensuring that each block represents an effectively independent sample and providing reliable standard errors of the mean. The same procedure was applied to assess the significance of disorder differences between the systems. Additionally, we calculated the SASA of phosphorylation-prone serine and threonine residues within the GPR183 C-terminal tail in simulations performed with and without explicit lipids. These analyses were carried out for both the inactive receptor ensemble and the active G protein–bound systems in WT and A338V variants. Differences in SASA between lipid-containing and lipid-free environments were subsequently used to quantify residue-specific lipid interactions and membrane burial, allowing assessment of how the A338V mutation alters membrane association of regulatory phosphorylation sites within the C-tail.

*Allosteric communication analysis*

Allosteric communication networks were constructed from the molecular dynamics trajectories using the AlloViz Python package.[16] AlloViz generates residue–residue interaction networks from MD simulations by quantifying correlated residue motions and applies graph-theoretical analyses to identify communication pathways and key residues involved in long-range allosteric signal propagation. The resulting communication networks were visualized and compared between the WT and A338V systems to identify mutation-induced changes in allosteric communication. In addition, the $C^{\alpha}$ distance between $R129^{3.50}$ (TM3) and $L243^{6.34}$ (TM6)[17, 18] was calculated throughout the trajectories to assess changes in the transmembrane core architecture in the inactive and active conformations of both the WT and A338V receptors as used[18] by us before. Statistical uncertainties were estimated by block averaging using a block size of 200 frames, ensuring that each block represented an effectively independent sample and providing reliable estimates of the standard error of the mean.

*Cell culture*

HEK293A cells and HL-60 cells were kept at 37°C with 5% $CO_2$in T-75 flasks. HEK293A cells were cultured in 10 ml DMEM, containing 10% heat-inactivated FBS (Sigma) and 1% PenStrep, (Sigma). HL-60 cells were cultured in 10 ml RPMI-1640 with 10% heat-inactivated FBS and 1% PenStrep and kept at density of below $10^6$ cells/mL. Culture media were changed every 2-4 days. The absence of mycoplasma contamination was routinely confirmed by MycoStrip's (InvivoGen) patented isothermal PCR detecting 16S ribosomal RNA of mycoplasma in the media after 2–3 days of cell exposure.

*DNA constructs, cloning and mutagenesis*

HiBiT-GPR183, HiBiT-GPR183-Nluc and HiBiT-GPR183-mNG-Nluc constructs were published by us before.[19] HiBiT-GPR183-cpGFP sensor was generated using Gibson cloning, replacing ICL3, i.e. residues between and including $Q229^{5.71}$ and $V238^{6.29}$, with cpGFP with linkers (LSSLI-cpGFP-NHDQL) from $FZD_5$-cpGFP.[20] Plasmid DNA constructs encoding the GPR183 A338V and Nluc R164Q (Nluc*) were generated with a GeneArt Site Directed mutagenesis kit (Thermo Fisher Scientific). rGFP-CAAX, Rap1Gap1a-*R*luc2 plasmid DNA and β-arrestin2-*R*luc2 in the pcDNA3.1(+) backbone were synthesized by GenScript. Venus-KRas, Nluc-mGsi and Venus-mGsi were kind gifts from Nevin Lambert (Augusta University). $FZD_5$-cpGFP, β-arrestin2-Venus, GRK2-Venus and GRK2-*R*luc2 were kind gifts from Gunnar Schulte (Karolinska Institutet). EPAC-based cAMP sensor H187 plasmid DNA[21] was a kind gift from Kees Jalink (The Netherlands Cancer Institute). Plasmid DNA encoding an α subunit of $Gi_1$ was from cDNA.org. Salmon sperm DNA (ss DNA) was from Thermo Fisher Scientific. The constructs were validated by Sanger sequencing (Eurofins GATC).

*Ligand*

The endogenous GPR183 agonist 7α,25-dihydroxycholesterol (#SML0541; referred also as the GPR183 agonist throughout the study) was from Sigma. The ligand was dissolved in DMSO at 5 mM, aliquoted, and stored at -20°C. Each aliquot was used a maximum of three times.

*Enhanced bystander BRET (ebBRET; Rluc2-rGFP) setup*

HEK293A cells were transiently transfected in suspension using polyethylenimine (PEI, Polysciences). To measure heterotrimeric Gi protein activation/dissociation at the cell membrane

interface, a total of ca. 4 × $10^5$ cells were transfected in 1 mL with 200 ng of HiBiT-GPR183 plasmid DNA constructs, 300 ng of rGFP-CAAX plasmid DNA, 100 ng of an α subunit of $Gi_1$ plasmid DNA, 50 ng of Rap1Gap-*R*luc2 plasmid DNA and 350 ng of ssDNA. To measure GRK2 recruitment to the cell membrane-expressed GPR183 in an ebBRET paradigm, a total of ca. 4 × $10^5$ cells in 1 mL were transfected with 200 ng of HiBiT-GPR183 plasmid DNA, 50 ng of GRK2-*R*luc2 plasmid DNA, 300 ng of rGFP-CAAX plasmid DNA and 450 ng of ssDNA. To measure β-arrestin2 recruitment to the cell membrane-expressed GPR183 in an ebBRET paradigm, a total of ca. 4 × $10^5$ cells in 1 mL were transfected with 200 ng of HiBiT-GPR183 plasmid DNA, 50 ng of β-arrestin2-*R*luc2 plasmid DNA, 300 ng of rGFP-CAAX plasmid DNA and 450 ng of ssDNA. Next, transfected cells (4 × $10^4$ cells in 100 µL) were seeded onto white 96-well cell culture plates. 24h later, the cells were washed once with 200 µL of HBSS (HyClone). Next, 80 µL of HBSS was added to the wells, and subsequently, 10 µL of furimazine (1:1000 final concentration, Promega) was added. The plate was incubated for 10 min, which was followed by the addition of 10 µL of the agonist. Next, *R*luc2 emission (donor, 360-440 nm, 100 ms integration time) and rGFP emission (acceptor, 505-575 nm, 100 ms integration time) were measured at 37°C (three measurements for baseline, 15 min with the agonist). The ebBRET ratios were defined as acceptor emission/donor emission. The measurements were performed using a Tecan Spark microplate reader.

*Nluc-Venus BRET setup*

HEK293A cells were transiently transfected in suspension using polyethylenimine (PEI, Polysciences). To measure miniGsi recruitment to the receptor, a total of ca. 4 × $10^5$ cells were transfected in 1 mL with 250 ng of Venus-mGsi plasmid DNA construct, 50 ng of HiBiT-GPR183-

Nluc plasmid DNA, 700 ng of ssDNA. To measure GRK2 recruitment to the receptor, a total of ca. $4 \times 10^5$ cells were transfected in 1 mL with 250 ng of GRK2-Venus plasmid DNA construct, 50 ng of HiBiT-GPR183-Nluc plasmid DNA, 700 ng of ssDNA. To measure β-arrestin2 recruitment to the receptor, a total of ca. $4 \times 10^5$ cells were transfected in 1 mL with 250 ng of β-arrestin2-Venus plasmid DNA construct, 50 ng of HiBiT-GPR183-Nluc plasmid DNA, 700 ng of ssDNA. To measure mGsi recruitment to the receptor at the plasma interface, a total of ca. $4 \times 10^5$ cells were transfected in 1 mL with 250 ng of Venus-Kras plasmid DNA construct, 10 ng of Nluc-mGsi plasmid DNA, 100 ng of HiBiT-GPR183 plasmid DNA and 640 ng of ssDNA. To receptor expression at the cell surface, a total of ca. $4 \times 10^5$ cells were transfected in 1 mL with 900 ng of Venus-Kras plasmid DNA construct and 100 ng of GPR183-Nluc. Next, transfected cells ($4 \times 10^4$ cells in 100 μL) were seeded onto white 96-well cell culture plates. 24h later, the cells were washed once with 200 μL of HBSS (HyClone). Next, 80 μL of HBSS was added to the wells, and subsequently, 10 μL of furimazine (1:1000 final concentration, Promega) was added. The plate was incubated for 10 min, which was followed by the addition of 10 μL of the agonist. Next, Nluc emission (donor, 460-500 nm, 50 ms integration time) and Venus emission (acceptor, 520-560 nm, 50 ms integration time) were measured at 37°C (three measurements for baseline, 15 min with the agonist). The BRET ratios were defined as acceptor emission/donor emission. The measurements were performed using a Tecan Spark microplate reader.

*BRET-based GPR183 conformational sensors experiments*

HEK293A cells were transiently transfected in suspension using PEI. To measure ligand-induced conformational change in GPR183 a total of ca. $4 \times 10^5$ cells were transfected in 1 mL with 50 ng of HiBiT-GPR183-mNG-Nluc plasmid DNA constructs and 950 ng of ssDNA. Next, transfected

cells ($4 \times 10^4$ cells in 100 µL) were seeded onto white 96-well cell culture plates. 24 h later, the cells were washed once with 200 µl of HBSS (HyClone). Next, 80 µL of HBSS was added to the wells, and subsequently, 10 µL of 1:1000 final furimazine (Promega) was added for the mNG-Nluc sensors. The plate was incubated for 10 min, which was followed by the ligand. Next, Nluc emission (donor, 460-500 nm, 50 ms integration time) and mNG emission (acceptor, 505-545 nm, 50 ms integration time) were measured at 37°C (three measurements for baseline, 15 min with agonis). The BRET ratio was defined as acceptor emission/donor emission. The measurements were performed using a Tecan Spark microplate reader.

*cpGFP-based GPR183 conformational biosensors experiments*

HEK293A cells were transiently transfected in suspension using Lipofectamine 2000. To measure agonist-induced conformational change in GPR183 a total of ca. $4 \times 10^5$ cells were transfected in 1 mL with 200 ng of HiBiT-GPR183-cpGFP WT or A338V plasmid DNA constructs and 800 ng of ssDNA. Next, transfected cells ($4 \times 10^4$ cells in 100 µL) were seeded onto black 96-well poly-D-lysine-coated cell culture plates. 24 h later, the cells were washed once with 200 µl of HBSS. Next, 90 µL of HBSS was added to the wells, and basal cpGFP fluorescence (470-15/515-20 nm, 40 flashes per well) was measured 5 times at 37°C. Subsequently, 10 µL of different concentrations of the agonist were added and the plate was measured for 15 min. The measurements were performed using a CLARIOstar plate reader (BMG).

*Luminescence-based measurements of cell surface expression of N-terminally HiBiT-tagged receptors*

HEK293A cells at a density of $4 \times 10^5$ cells/mL were transfected in suspension. HEK293A cells were transfected using PEI with 200 ng of the indicated receptor plasmid DNA (HiBiT-GPR183

wild type (WT) or A338V and with or without Nluc or cpGFP insertions) and 800 ng ssDNA. HEK293A cells in a volume of 100 µL were seeded onto a white 96-well plate or a black 96-well plate (cpGFP conformational sensors). 24 h post-transfection, the cells were washed once in HBSS and incubated with 100 µL of furimazine/LgBiT mix (1:100 final concentration for furimazine and 1:200 final concentration LgBiT, Promega) for 10 min. Subsequently Nluc/NanoBiT luminescence (460-500 nm, 50 ms integration time) was measured using a Tecan Spark microplate reader.

*FRET-based cAMP production*

HEK293A cells were transiently transfected in suspension using Lipofectamine 2000 (Thermo Fisher Scientific). A total of ca. $4 \times 10^5$ cells were transfected in 1 ml with 200 ng of HiBiT-GPR183 constructs or ssDNA, 100 ng EPAC-based cAMP sensor and 700 ng of ssDNA plasmid DNA. Next, transfected cells ($4 \times 10^4$ cells in 100 µl) were seeded onto black 96-well cell culture plates. Twenty-four hours later, the cells were washed once with 200 µl of HBSS (HyClone). Next, 90 µl of HBSS was added to the wells and FRET ratio measured was measured in three consecutive reads with a CLARIOstar plate reader (BMG) at room temperature. mTurquoise2 (donor) was excited at 410-450 nm and its emission intensity using a 460-500 nm monochromator. cpVenus173 (acceptor) emission was recorded using a 515-555 nm monochromator. Next, different concentrations of 7α,25-dihydroxycholesterol together with 2.7 µM forskolin (Sigma) were added (10 µL) and the measurements continued for 15 min. FRET ratios were defined as acceptor emission/donor emission. Here, an increase in FRET indicates a decrease in cAMP levels.

*Internalization assay*

HEK293A cells at a density of 4 x $10^5$ cells/mL were transfected in suspension with PEI with 200 ng of the indicated receptor plasmid DNA (HiBiT-GPR183 wild type (WT) or A338V. Next, transfected cells (4 × $10^4$ cells in 100 μL) were seeded onto white 96-well cell culture plates. 24 h later, the cells were washed once with 200 μl of HBSS (HyClone). Next, 80 μL of HBSS was added to the wells, and subsequently, 10 μL of the final concentration of 1 μM of the agonist was added every 10 min for 70 min. The plate was kept at 37°C. Furimazine was added to all the wells together with the last ligand addition. Next, Nluc emission (donor, 460-500 nm, 50 ms integration time) was measured at 37°C (three measurements). The measurements were performed using a Tecan Spark microplate reader.

*Migration of neutrophil-differentiated HL-60 cells*

1 x $10^5$/mL of HL-60 were differentiated into neutrophils using treatment with 1% DMSO and 1 μM All-trans retinoic acid (ATRA) (Sigma) for five days. The differentiated cells were electroporated at 20 x $10^6$ cells/ml in 100 μL with 1 μg of HiBiT-GPR183 WT or A338V plasmid DNA using Ingenio® EZporator® Pulse generator at 200 V in a low voltage mode. The electroporated and cells were seeded onto a 24-well plate (2x$10^6$ cells/well in 1 mL). On the next day, small aliquots were taken to measure luminescence indicative of cell surface expression (as above for HEK293A cells), and the rest of the cells were washed and labelled for 30 min with 3 μM calcein (Sigma) and used in a 180 min migration assay through 5 μm membrane towards the agonist/chemoattractant 7α,25-dihydroxycholesterol at 10 nM or vehicle. The cells from the upper and lower chambers were subsequently transferred to separate wells of a black 96-well plate and the calcein fluorescence (485-20/535-25 nM) measured on a Tecan Spark microplate reader.

*Immunoblotting*

HEK293A cells were transfected at a density of 4 × $10^5$ cells, with a total volume of 1 mL used per transfection reaction. The DNA mix for each transfection included pcDNA, HiBiT-GPR183 WT or HiBiT-GPR183 A338V. Specifically, the plasmid DNA mixes were composed of 200 ng of receptor plasmid DNA and 800 ng of pcDNA, or just pcDNA for the control. The cells were incubated at 37°C for 24 hours. Whole-cell lysates were prepared by combining 50 µl of 2x Laemmli buffer containing 10% dithiothreitol with 50 µl of PBS. This mixture was subjected to sonication at maximum power in three sequences of 3-second bursts, each separated by 3-second pauses. The lysates were subsequently analyzed using 4–20% Mini-PROTEAN TGX precast polyacrylamide gels (Bio-Rad) along with the PageRuler Plus Prestained Protein Ladder (Thermo Fisher Scientific). The membranes were blocked with 5% non-fat dried milk in a Tris/NaCl/Tween20 buffer (TBS-T) and then incubated overnight at 4°C with primary antibodies diluted in the blocking buffer. The primary antibodies used were rabbit monoclonal anti-GAPDH (14C10; 1:2500 dilution; Cell Signaling Technology #2118) and mouse monoclonal anti-HiBiT (1.0 µg/ml; Promega clone 30E5; IgG2c with kappa light chain). Following several washes with TBS-T, the membranes were incubated with horseradish peroxidase-conjugated secondary antibodies (goat anti-mouse, 1:3000, Thermo Fisher Scientific #31430 and goat anti-rabbit, 1:3000, Thermo Fisher Scientific #31460) diluted in blocking buffer for 1 hour at room temperature. After additional washes, the membranes were exposed to Clarity Western ECL Blotting Substrate (Bio-Rad) for 2 minutes. Protein bands were then visualized using the ChemiDoc system (Bio-Rad) and analyzed in ImageLab.

*Statistical analysis*

All data presented in the main figures are based on at least three independent experiments (biological replicates), with each individual experiment typically performed at least in duplicates (technical replicates) for each condition, unless otherwise specified in a figure legend. One biological replicate refers to wells containing cells seeded from the same individual cell culture flasks and measured on the same day. Different biological replicates were transfected using separate transfection mixtures and measured on different days. Technical replicates are defined as individual wells with cells from the same biological replicate. Samples were not randomized or blinded during the experiments. Molecular dynamics trajectory analyses and visualization were performed using cpptraj, DSSP, and custom Python scripts employing NumPy, SciPy, Matplotlib, and Biopython libraries. Concentration response data were fitted to either a three-parameter or a four-parameter non-linear model selected with the extra sum-of-squares F-test. Two datasets were analyzed for statistical differences with *t*-test. Three or more datasets were analyzed by one-way ANOVA with multiple comparison Dunnett's post-hoc analysis. Significance levels are given and displayed in the figures as: $^{*}P < 0.05$; $^{**}P < 0.01$; $^{***}P < 0.001$; $^{****}P < 0.0001$. For the in vitro assays, exact P values are provided either in the text or in the corresponding figures. Moreover, where significant differences were observed, concentration–response data are reported in the text together with the corresponding 95% confidence intervals. Differences between datasets that did not reach statistical significance are left unmarked. Data points presented in the concentration response plots throughout the manuscript are indicated as the mean ± standard deviation (s.d.) and the data represented as dot plots are indicated the mean ± standard error of the mean (s.e.m.) unless otherwise stated in a figure legend. Concentration response curves are presented as net AUC from the kinetic curves from the respective experiments. First, we obtain %ΔBRET or %ΔFRET or

Δ fluorescence (for the cpGFP sensors) for each well and for each measurement upon applying baseline correction: 100*(value-baseline)/baseline, where baseline is defined as an average BRET or FRET from three BRET/FRET measurements or average fluorescence from five fluorescence measurements prior to the agonist addition. Subsequently, to show agonist-treated data in relation to the vehicle-treated data, vehicle %ΔBRET or %ΔFRET or %Δ fluorescence data were subtracted from %ΔBRET or %ΔFRET or %Δ fluorescence for each agonist concentration. Using these baseline-corrected, vehicle-corrected %ΔBRET/%ΔFRET/%Δ fluorescence vs time kinetics plots, net AUC (negative peaks are subtracted from positive peaks) is calculated in GraphPad Prism 11.0.0. All the other statistical and graphical analyses for experimental data were also performed using this version of GraphPad Prism software.

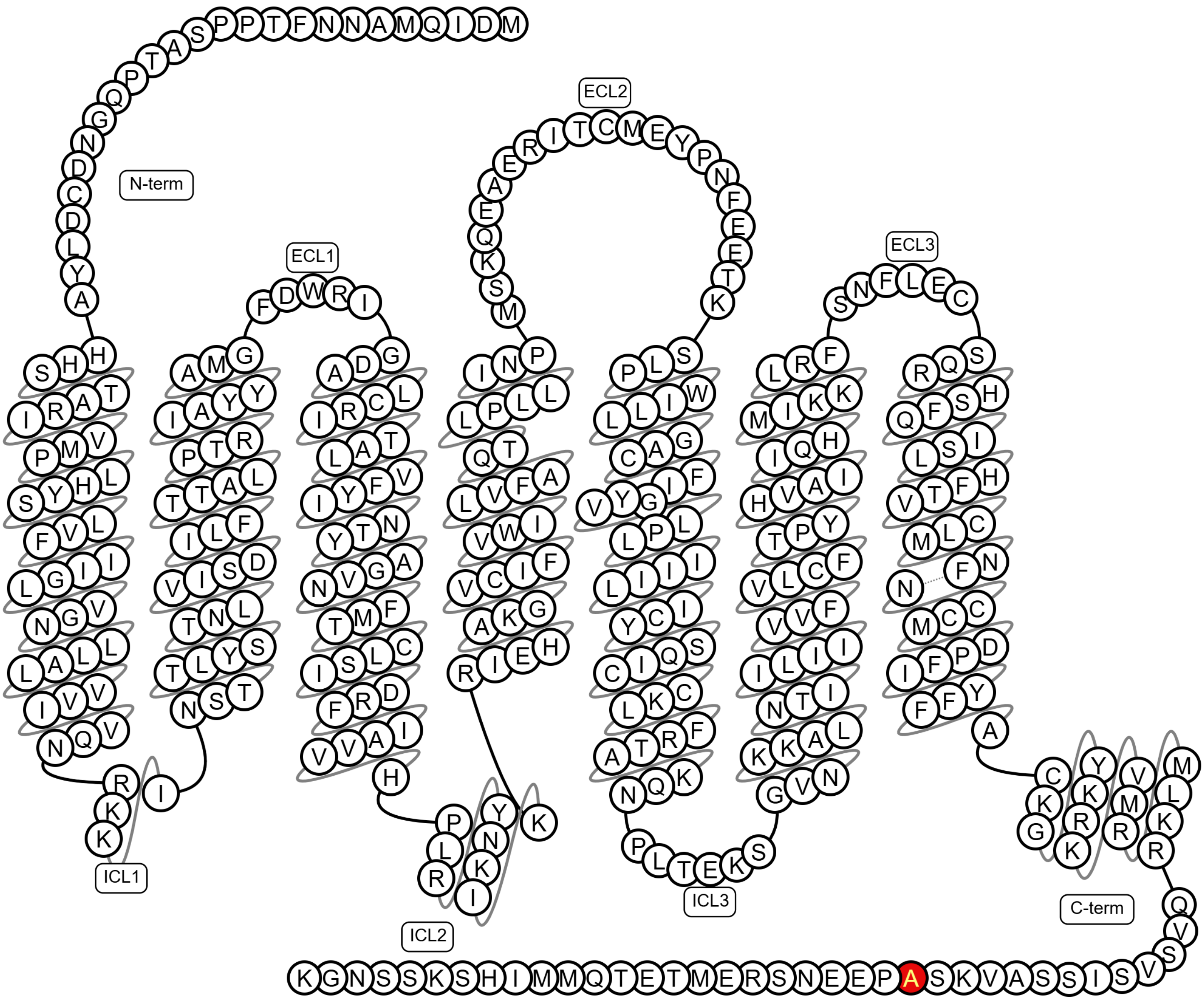
N-term
ECL1
ECL2
ECL3
ICL1
ICL2
ICL3
C-term

**Supplementary Figure 1. Schematic representation of the human GPR183 with alanine at a position 338 (A338V) shown in red.**

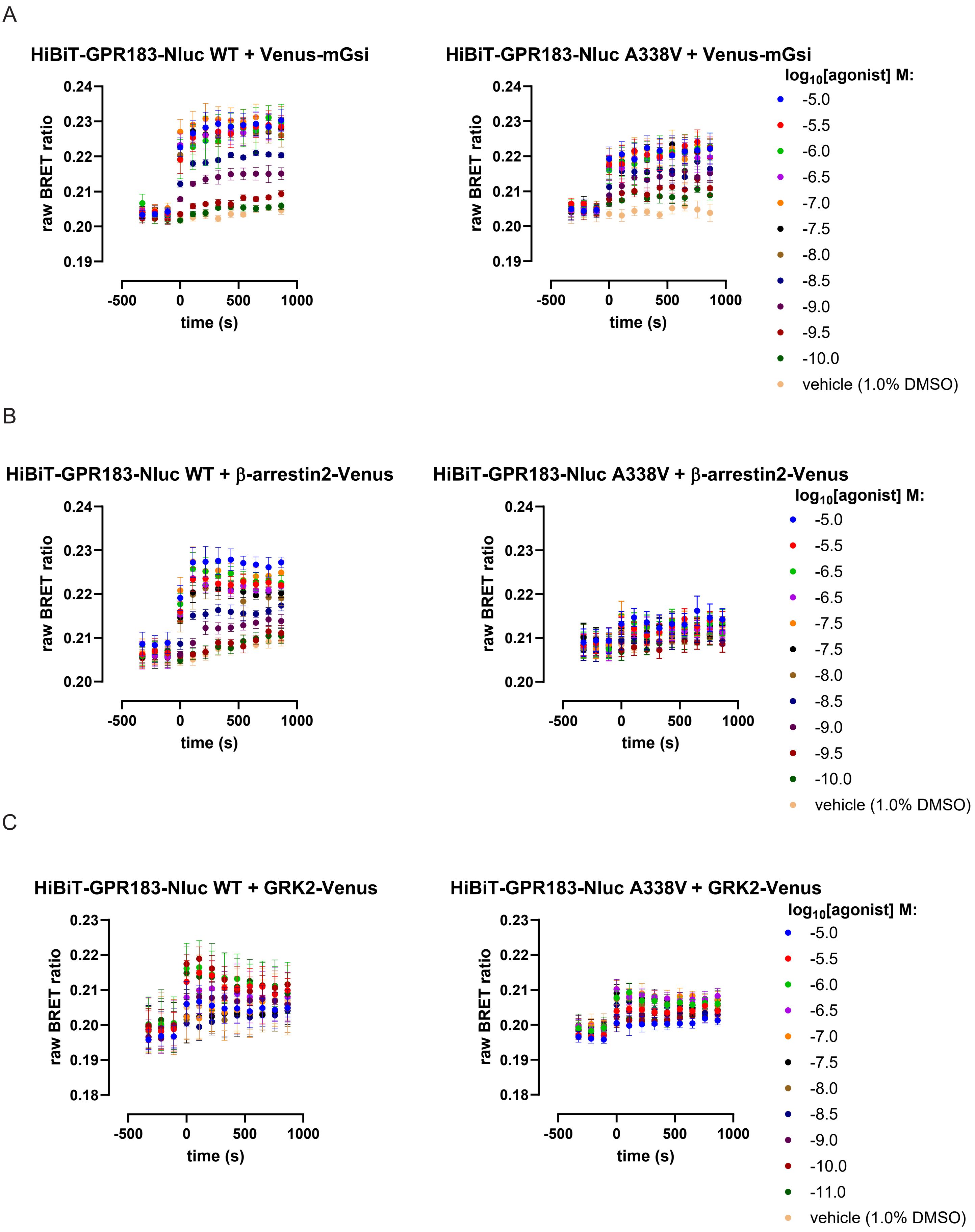
A
HiBiT-GPR183-Nluc WT + Venus-mGsi
HiBiT-GPR183-Nluc A338V + Venus-mGsi
raw BRET ratio
0.24
0.23
0.22
0.21
0.20
0.19
-500
0
500
1000
time (s)
log10[agonist] M:
-5.0
-5.5
-6.0
-6.5
-7.0
-7.5
-8.0
-8.5
-9.0
-9.5
-10.0
vehicle (1.0% DMSO)
B
HiBiT-GPR183-Nluc WT + β-arrestin2-Venus
HiBiT-GPR183-Nluc A338V + β-arrestin2-Venus
raw BRET ratio
0.24
0.23
0.22
0.21
0.20
-500
0
500
1000
time (s)
log10[agonist] M:
-5.0
-5.5
-6.5
-6.5
-7.5
-7.5
-8.0
-8.5
-9.0
-9.5
-10.0
vehicle (1.0% DMSO)
C
HiBiT-GPR183-Nluc WT + GRK2-Venus
HiBiT-GPR183-Nluc A338V + GRK2-Venus
raw BRET ratio
0.23
0.22
0.21
0.20
0.19
0.18
-500
0
500
1000
time (s)
log10[agonist] M:
-5.0
-5.5
-6.0
-6.5
-7.0
-7.5
-8.0
-8.5
-9.0
-10.0
-11.0
vehicle (1.0% DMSO)

**Supplementary Figure 2. A.** BRET kinetic traces of direct BRET assay measuring Venus-mGsi recruitment to receptor-Nluc, agonist was added at time = 0. **B.** BRET kinetic traces of direct BRET assay measuring β-arrestin2-Venus recruitment to receptor-Nluc, agonist was added at time = 0. **C.** BRET kinetic traces of direct BRET assay measuring GRK2-Venus recruitment to receptor-Nluc, agonist was added at time = 0. Kinetic traces are represented as mean ± s.e.m. of n≥3 independent experiments, which were performed at least in technical duplicates each. Data are represented as raw BRET counts.

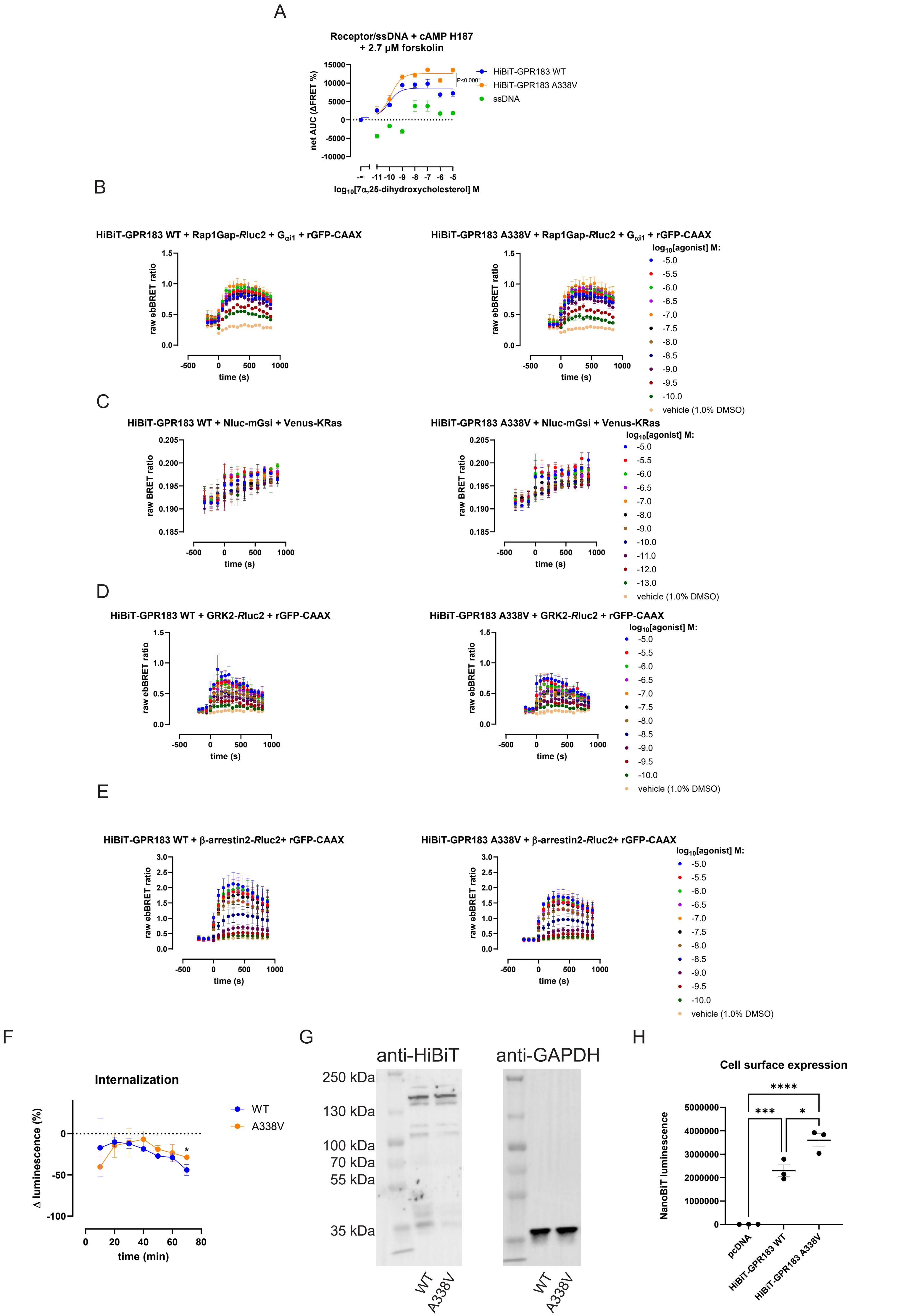
A
Receptor/ssDNA + cAMP H187
+ 2.7 µM forskolin
net AUC (ΔFRET %)
15000
10000
5000
0
-5000
-10000
P<0.0001
HiBiT-GPR183 WT
HiBiT-GPR183 A338V
ssDNA
-∞ -11 -10 -9 -8 -7 -6 -5
log10[7α,25-dihydroxycholesterol] M
B
HiBiT-GPR183 WT + Rap1Gap-Rluc2 + Gαi1 + rGFP-CAAX
HiBiT-GPR183 A338V + Rap1Gap-Rluc2 + Gαi1 + rGFP-CAAX
raw ebBRET ratio
1.5
1.0
0.5
0.0
-500
0
500
1000
time (s)
log10[agonist] M:
-5.0
-5.5
-6.0
-6.5
-7.0
-7.5
-8.0
-8.5
-9.0
-9.5
-10.0
vehicle (1.0% DMSO)
C
HiBiT-GPR183 WT + Nluc-mGsi + Venus-KRas
HiBiT-GPR183 A338V + Nluc-mGsi + Venus-KRas
raw BRET ratio
0.205
0.200
0.195
0.190
0.185
time (s)
log10[agonist] M:
-5.0
-5.5
-6.0
-6.5
-7.0
-8.0
-9.0
-10.0
-11.0
-12.0
-13.0
vehicle (1.0% DMSO)
D
HiBiT-GPR183 WT + GRK2-Rluc2 + rGFP-CAAX
HiBiT-GPR183 A338V + GRK2-Rluc2 + rGFP-CAAX
raw ebBRET ratio
time (s)
log10[agonist] M:
-5.0
-5.5
-6.0
-6.5
-7.0
-7.5
-8.0
-8.5
-9.0
-9.5
-10.0
vehicle (1.0% DMSO)
E
HiBiT-GPR183 WT + β-arrestin2-Rluc2+ rGFP-CAAX
HiBiT-GPR183 A338V + β-arrestin2-Rluc2+ rGFP-CAAX
raw ebBRET ratio
3.0
2.5
2.0
1.5
1.0
0.5
0.0
time (s)
log10[agonist] M:
-5.0
-5.5
-6.0
-6.5
-7.0
-7.5
-8.0
-8.5
-9.0
-9.5
-10.0
vehicle (1.0% DMSO)
F
Internalization
Δ luminescence (%)
0
-50
-100
0 20 40 60 80
time (min)
WT
A338V
*
G
anti-HiBiT
anti-GAPDH
250 kDa
130 kDa
100 kDa
70 kDa
55 kDa
35 kDa
WT
A338V
WT
A338V
H
Cell surface expression
NanoBiT luminescence
5000000
4000000
3000000
2000000
1000000
0
****
***
*
pcDNA
HiBiT-GPR183 WT
HiBiT-GPR183 A338V

**Supplementary Figure 3. A.** Agonist stimulation of HiBiT-GPR183 A338V leads to significantly lower levels of cAMP in forskolin-stimulated HEK293A cells. Data are presented as net AUC of ΔFRET % from pooled n=4 independent experiments. Data are presented as mean ± s.d. In this setup, an increase in FRET is indicative of a decrease in cAMP levels. **B.** ebBRET kinetic traces of ebBRET assay measuring activation of Gi, agonist was added at time = 0. **C.** BRET kinetic traces of bystander BRET assay measuring Nluc-mGsi recruitment to untagged membrane-bound receptor, agonist was added at time = 0. **D.** ebBRET kinetic traces of ebBRET assay measuring GRK2-*R*luc2 recruitment to membrane-bound receptor, agonist was added at time = 0. **E.** ebBRET kinetic traces of ebBRET assay measuring β-arrestin2-*R*luc2 recruitment to membrane-bound receptor, agonist was added at time = 0. Kinetic traces are represented as mean ± s.e.m. of n≥3 independent experiments, which were performed at least in technical duplicates each. Data are represented as raw BRET counts. **F.** Internalization assay. HiBiT-GPR183 WT or HiBiT-GPR183 A338V overexpressing HEK293A were treated with vehicle or 1 μM agonist every ten minutes for 70 min. The data are represented as Δ luminescence % between vehicle and agonist-treated wells for the same time point. Data come from n=4 independent experiments and are shown as mean ± s.e.m. **G.** Immunoblotting data showing total cell expression of HiBiT-GPR183 WT or A338V. Membranes were stained with anti-HiBiT and anti-GAPDH antibodies. The data presented here come from a representative experiment from three independent experiments. Predicted molecular weight of HiBiT-GPR183 = 42.5 kDa. **H.** Cell surface expression of HiBiT-GPR183 WT or A338V in live HEK293A cells. The data are represented as mean ± s.e.m. of n=3 independent experiments; pcDNA-transfected cells were used as a control.

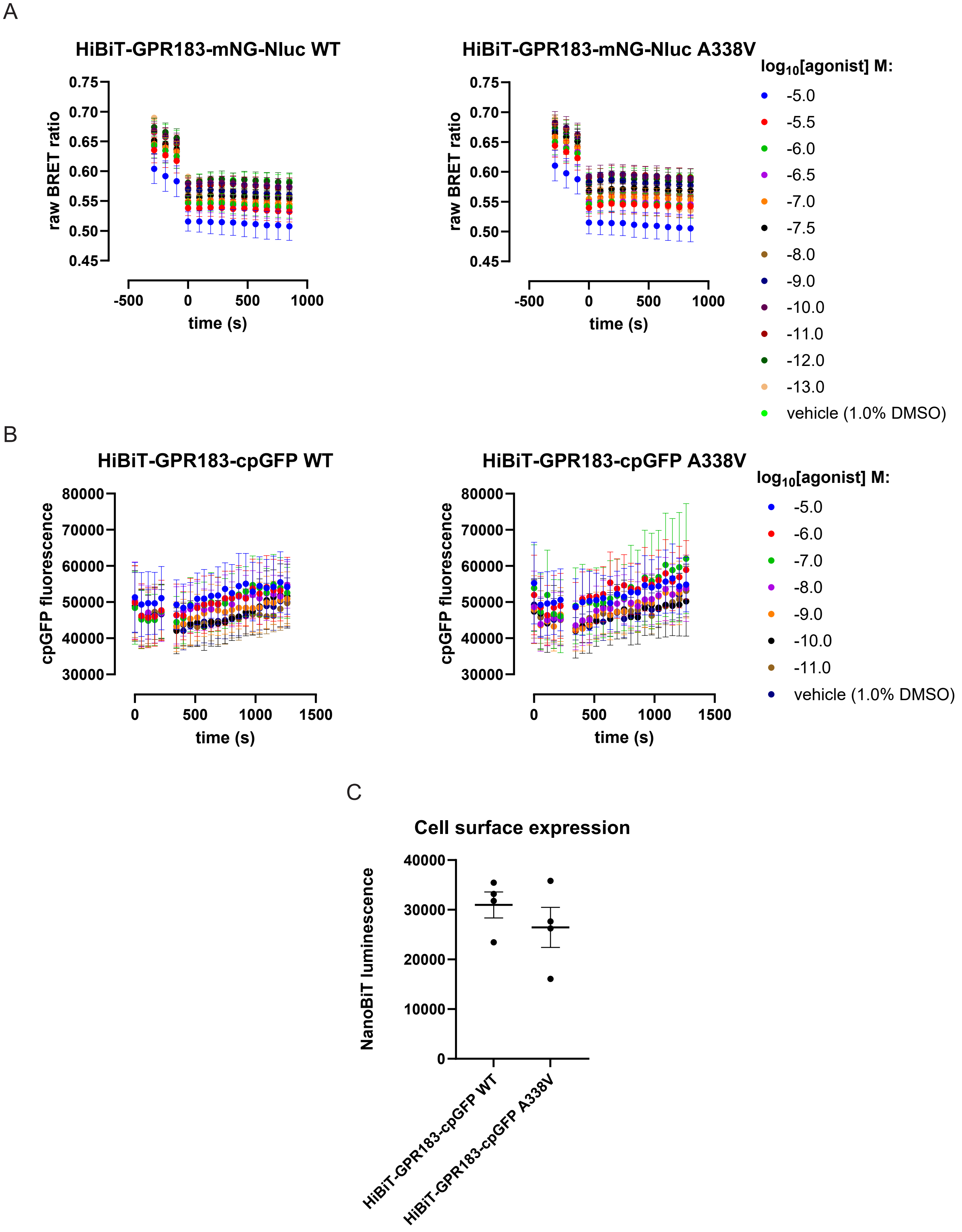
A
HiBiT-GPR183-mNG-Nluc WT
HiBiT-GPR183-mNG-Nluc A338V
raw BRET ratio
0.75
0.70
0.65
0.60
0.55
0.50
0.45
-500
0
500
1000
time (s)
log10[agonist] M:
-5.0
-5.5
-6.0
-6.5
-7.0
-7.5
-8.0
-9.0
-10.0
-11.0
-12.0
-13.0
vehicle (1.0% DMSO)
B
HiBiT-GPR183-cpGFP WT
HiBiT-GPR183-cpGFP A338V
cpGFP fluorescence
80000
70000
60000
50000
40000
30000
0
500
1000
1500
time (s)
log10[agonist] M:
-5.0
-6.0
-7.0
-8.0
-9.0
-10.0
-11.0
vehicle (1.0% DMSO)
C
Cell surface expression
NanoBiT luminescence
40000
30000
20000
10000
0
HiBiT-GPR183-cpGFP WT
HiBiT-GPR183-cpGFP A338V

**Supplementary Figure 4. A.** BRET kinetic traces of conformational mNG-Nluc sensors analysis, agonist was added at time = 0. **B.** Fluorescence kinetic traces of conformational cpGFP sensors, agonist was added after the 5$^{th}$ time point. **C.** Cell surface expression of HiBiT-GPR183-cpGFP WT or A338V in live HEK293A cells. The data are represented as mean ± s.e.m. of n=4 independent experiments.

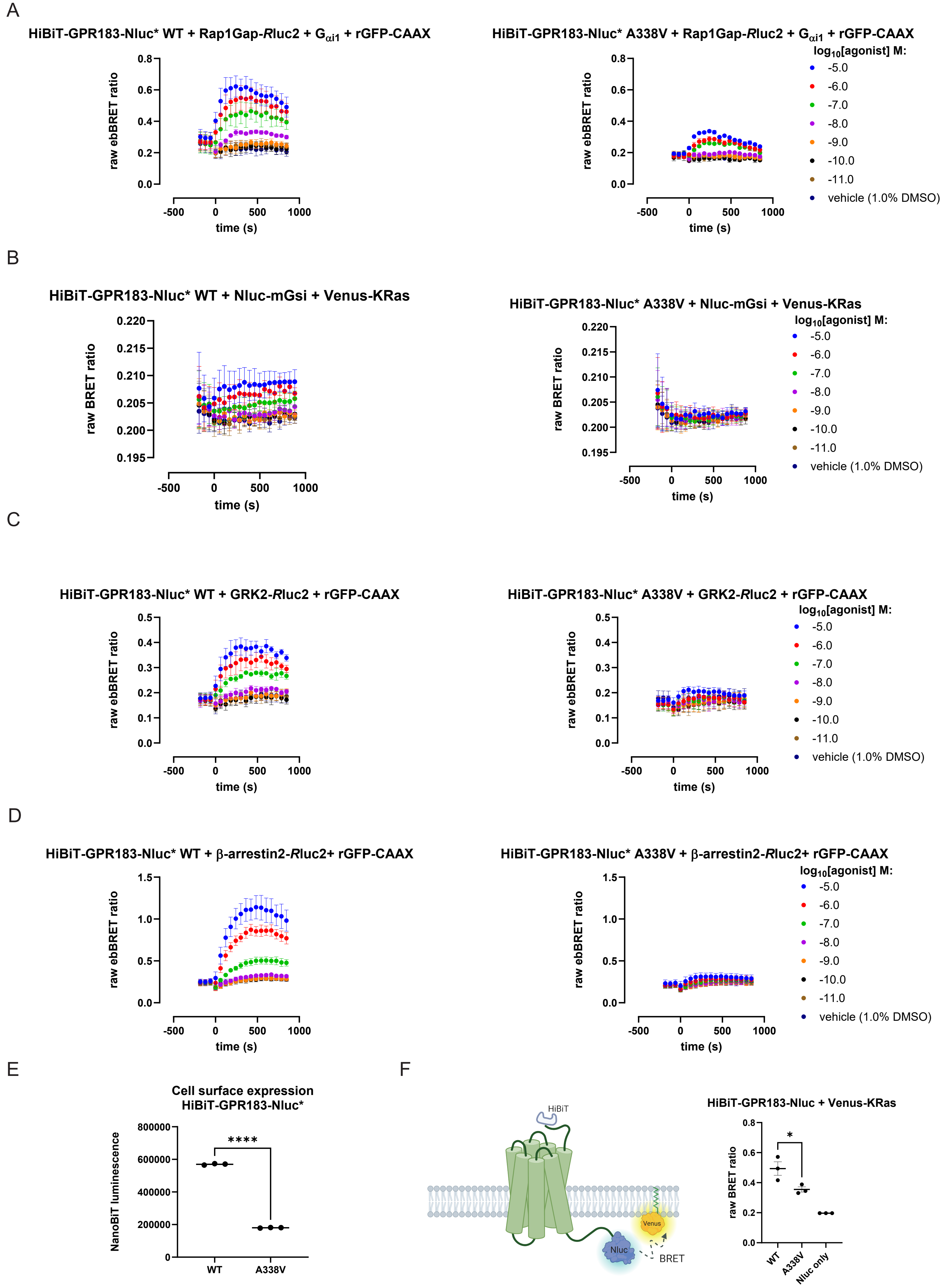

A
HiBiT-GPR183-Nluc* WT + Rap1Gap-Rluc2 + Gαi1 + rGFP-CAAX
raw ebBRET ratio
time (s)
HiBiT-GPR183-Nluc* A338V + Rap1Gap-Rluc2 + Gαi1 + rGFP-CAAX
log10[agonist] M:
-5.0
-6.0
-7.0
-8.0
-9.0
-10.0
-11.0
vehicle (1.0% DMSO)
B
HiBiT-GPR183-Nluc* WT + Nluc-mGsi + Venus-KRas
raw BRET ratio
time (s)
HiBiT-GPR183-Nluc* A338V + Nluc-mGsi + Venus-KRas
C
HiBiT-GPR183-Nluc* WT + GRK2-Rluc2 + rGFP-CAAX
HiBiT-GPR183-Nluc* A338V + GRK2-Rluc2 + rGFP-CAAX
D
HiBiT-GPR183-Nluc* WT + β-arrestin2-Rluc2+ rGFP-CAAX
HiBiT-GPR183-Nluc* A338V + β-arrestin2-Rluc2+ rGFP-CAAX
E
Cell surface expression
HiBiT-GPR183-Nluc*
NanoBiT luminescence
****
WT
A338V
F
HiBiT
Nluc
Venus
BRET
HiBiT-GPR183-Nluc + Venus-KRas
raw BRET ratio
*
WT
A338V
Nluc only

**Supplementary Figure 5. A.** ebBRET kinetic traces of ebBRET assay measuring activation of Gi in the setup with Receptor-Nluc*, agonist was added at time = 0. **B.** BRET kinetic traces of bystander BRET assay measuring Nluc-mGsi recruitment to membrane-bound Receptor-Nluc*, agonist was added at time = 0. **C.** ebBRET kinetic traces of ebBRET assay measuring GRK2-*R*luc2 recruitment to membrane-bound receptor-Nluc*, agonist was added at time = 0. **D.** ebBRET kinetic traces of ebBRET assay measuring β-arrestin2-*R*luc2 recruitment to membrane-bound receptor-Nluc*, agonist was added at time = 0. Kinetic traces are represented as mean ± s.e.m. of n≥3 independent experiments, which were performed at least in technical duplicates each. Data are represented as raw BRET counts. **E.** Cell surface expression of HiBiT-GPR183-Nluc* WT or A338V in live cells. The data are represented as mean ± s.e.m. of n=3 independent experiments. **F. Left:** Schematic representation of the experimental setup to assess cell surface expression by measuring BRET between the C-terminally Nluc-tagged receptor and cell membrane-inserted Venus. This setup would also measure differences in Nluc tag positioning in relation to the fixed Venus. **Right:** Bystander BRET data from experiments where the HEK293A cells were transfected with fixed amounts of the receptor plasmid DNA construct and Venus-KRas construct. The data are shown as bystander BRET between Nluc and Venus-Kras. Differences in BRET between WT and A338V can also indicate differences in the Nluc position in relation to the membrane-anchored Venus. The data are represented as mean ± s.e.m. of n=3 independent experiments; Nluc-DVL2 only was used as a control to measure background BRET in the absence of an energy acceptor.

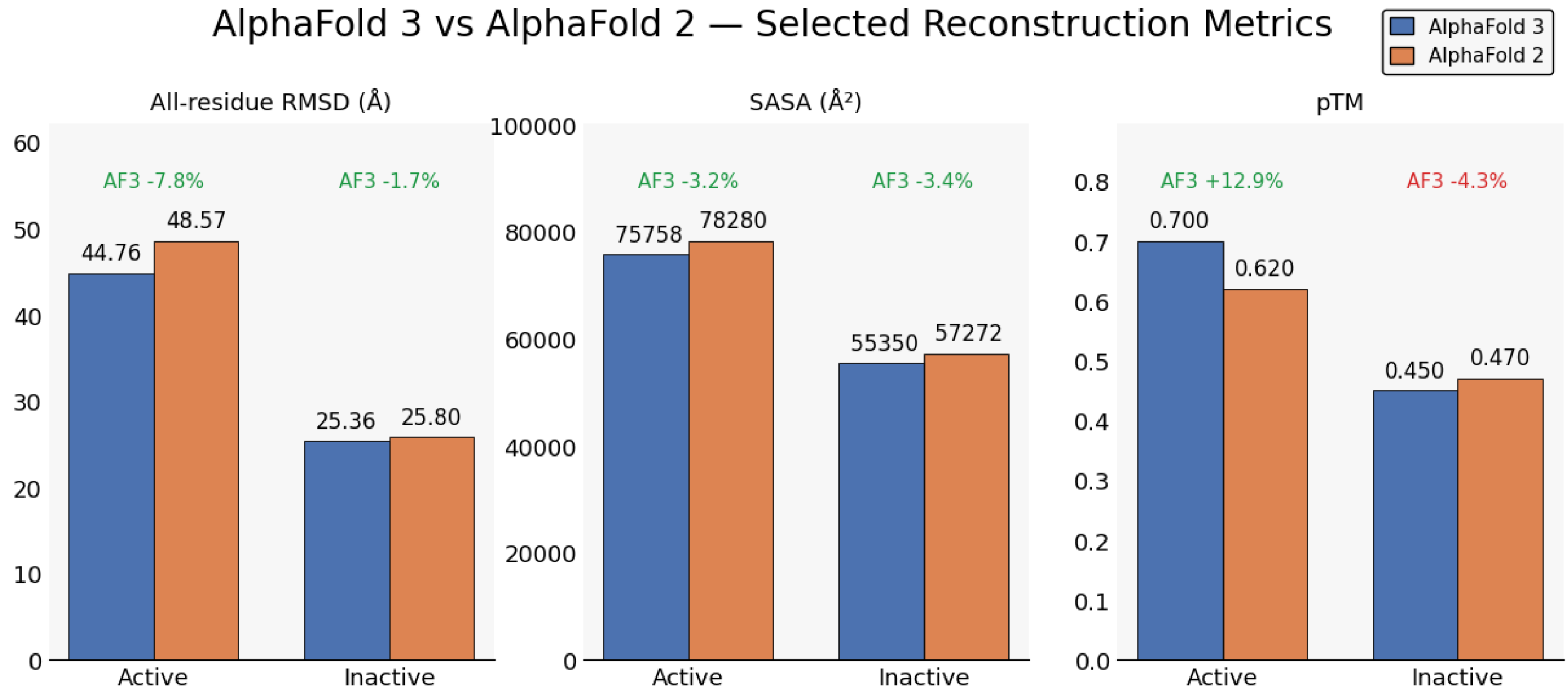

AlphaFold 3 vs AlphaFold 2 — Selected Reconstruction Metrics
AlphaFold 3
AlphaFold 2
All-residue RMSD (Å)
AF3 -7.8%
AF3 -1.7%
44.76
48.57
25.36
25.80
0
10
20
30
40
50
60
Active
Inactive
SASA (Å²)
AF3 -3.2%
AF3 -3.4%
75758
78280
55350
57272
0
20000
40000
60000
80000
100000
Active
Inactive
pTM
AF3 +12.9%
AF3 -4.3%
0.700
0.620
0.450
0.470
0.0
0.1
0.2
0.3
0.4
0.5
0.6
0.7
0.8
Active
Inactive

**Supplementary Figure 6. The comparison of RMSD, SASA and pTM metrics for the quality of predictions of AlphaFold 3 and AlphaFold 2.** Overall, AlphaFold3 provided the most accurate reconstruction of GPR183, yielding lower RMSD values with respect to the experimental structures relative to AlphaFold2, particularly for the active-state receptor. Although AlphaFold2 showed marginally improved radius of gyration and slightly lower C-terminal SASA values, these differences were minor, while both methods demonstrated reduced confidence for the inactive-state reconstruction, likely due to the presence of irregular Fab-associated regions.

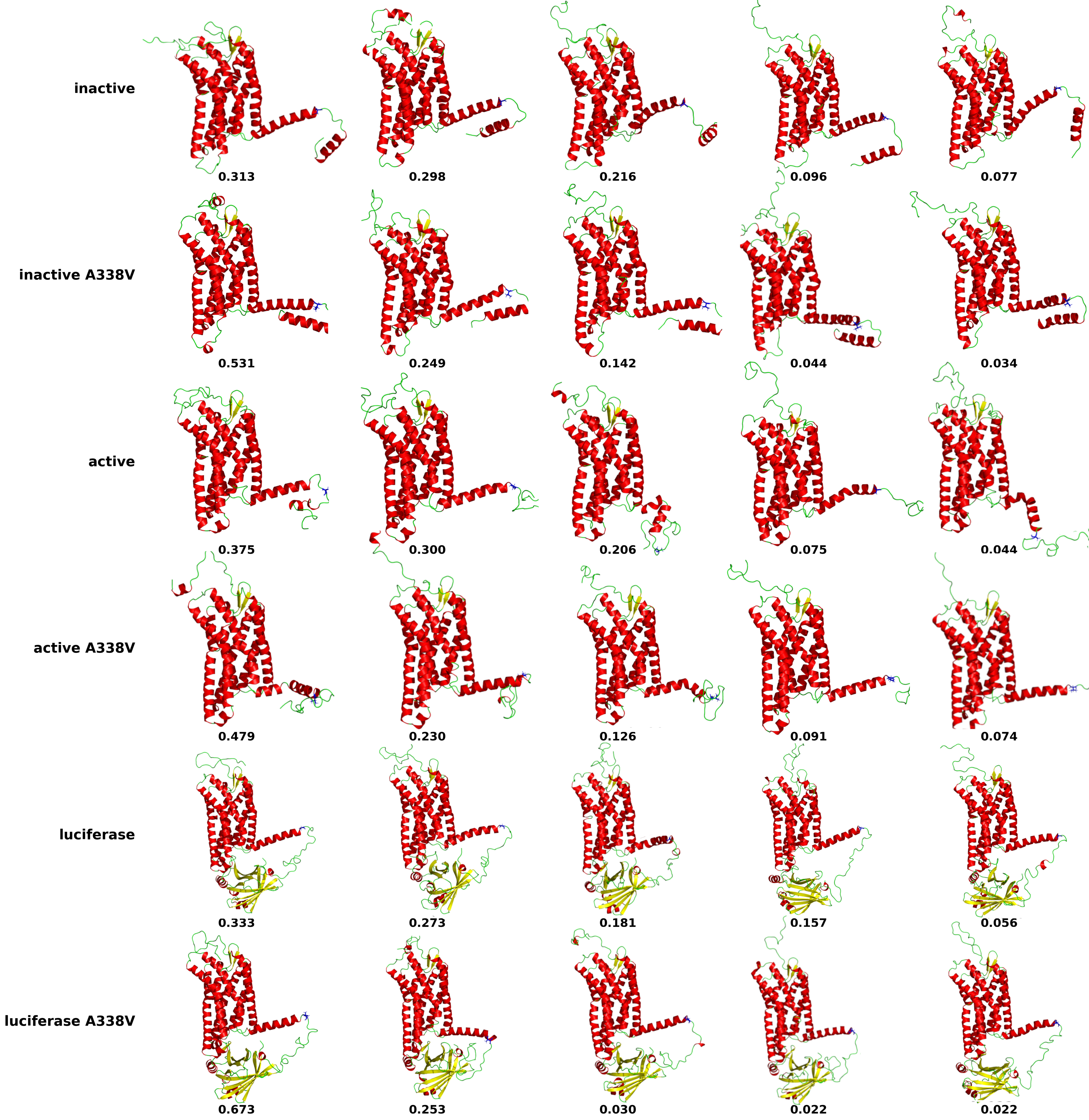
inactive
0.313
0.298
0.216
0.096
0.077
inactive A338V
0.531
0.249
0.142
0.044
0.034
active
0.375
0.300
0.206
0.075
0.044
active A338V
0.479
0.230
0.126
0.091
0.074
luciferase
0.333
0.273
0.181
0.157
0.056
luciferase A338V
0.673
0.253
0.030
0.022
0.022

**Supplementary Figure 7. The cluster populations highlight differences in the conformational ensembles across systems and mutation states.** The five most populated structural clusters extracted from the MD trajectories for each system. Representative structures are shown together with their relative populations within the simulated conformational ensembles.

A

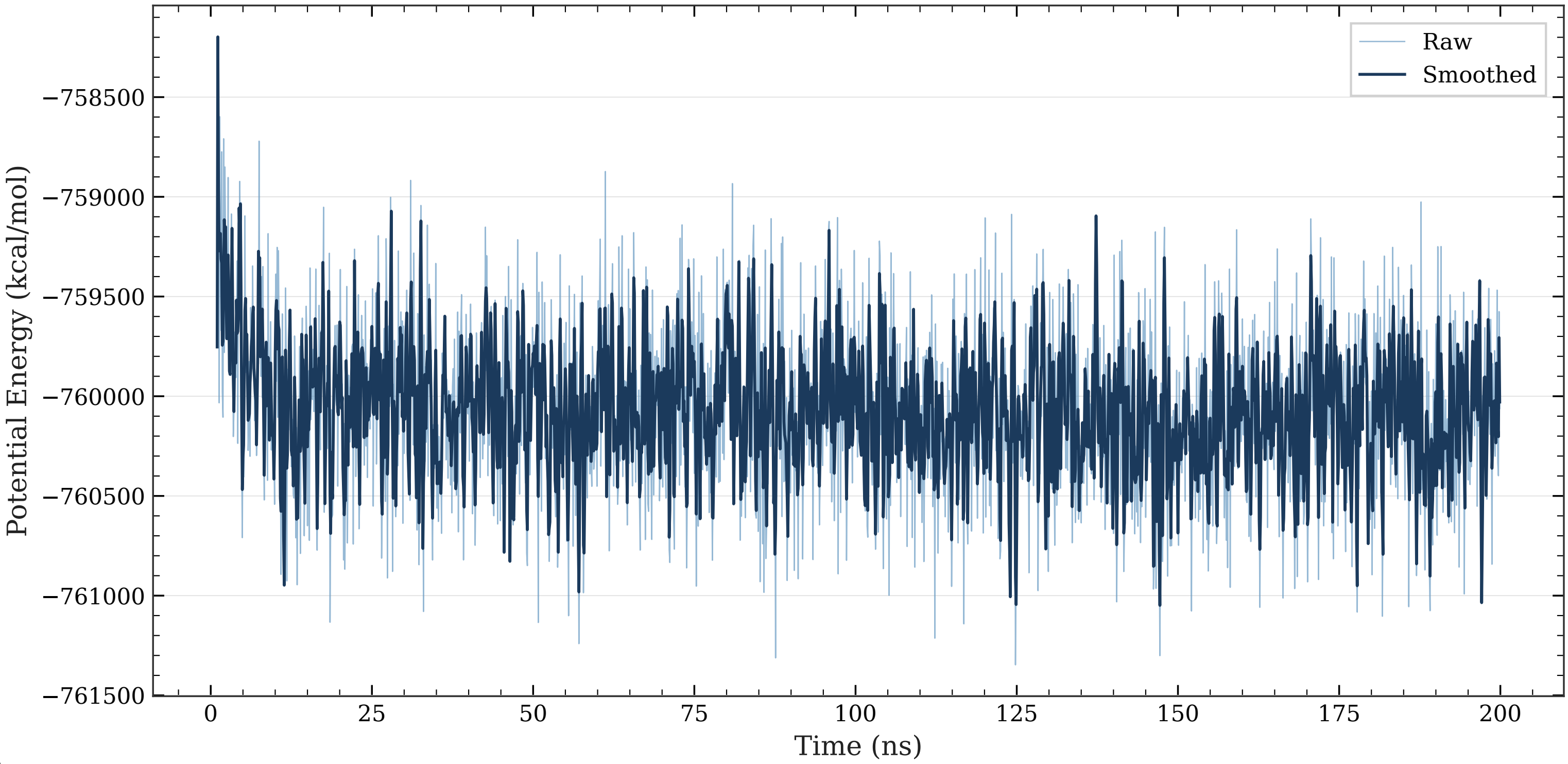

Raw
Smoothed
−758500
−759000
−759500
−760000
−760500
−761000
−761500
Potential Energy (kcal/mol)
0
25
50
75
100
125
150
175
200
Time (ns)


B

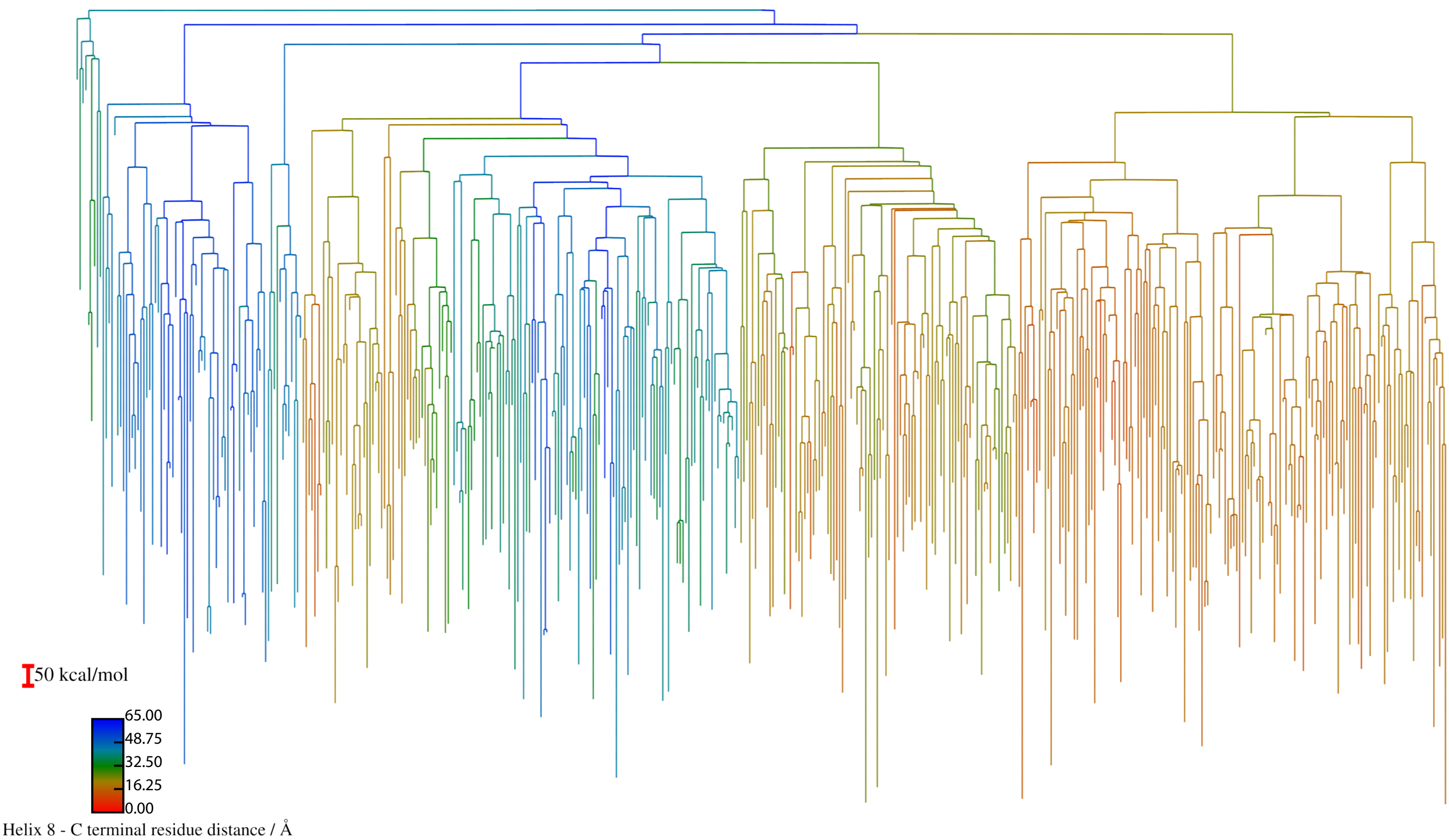

50 kcal/mol
65.00
48.75
32.50
16.25
0.00
Helix 8 - C terminal residue distance / Å

A

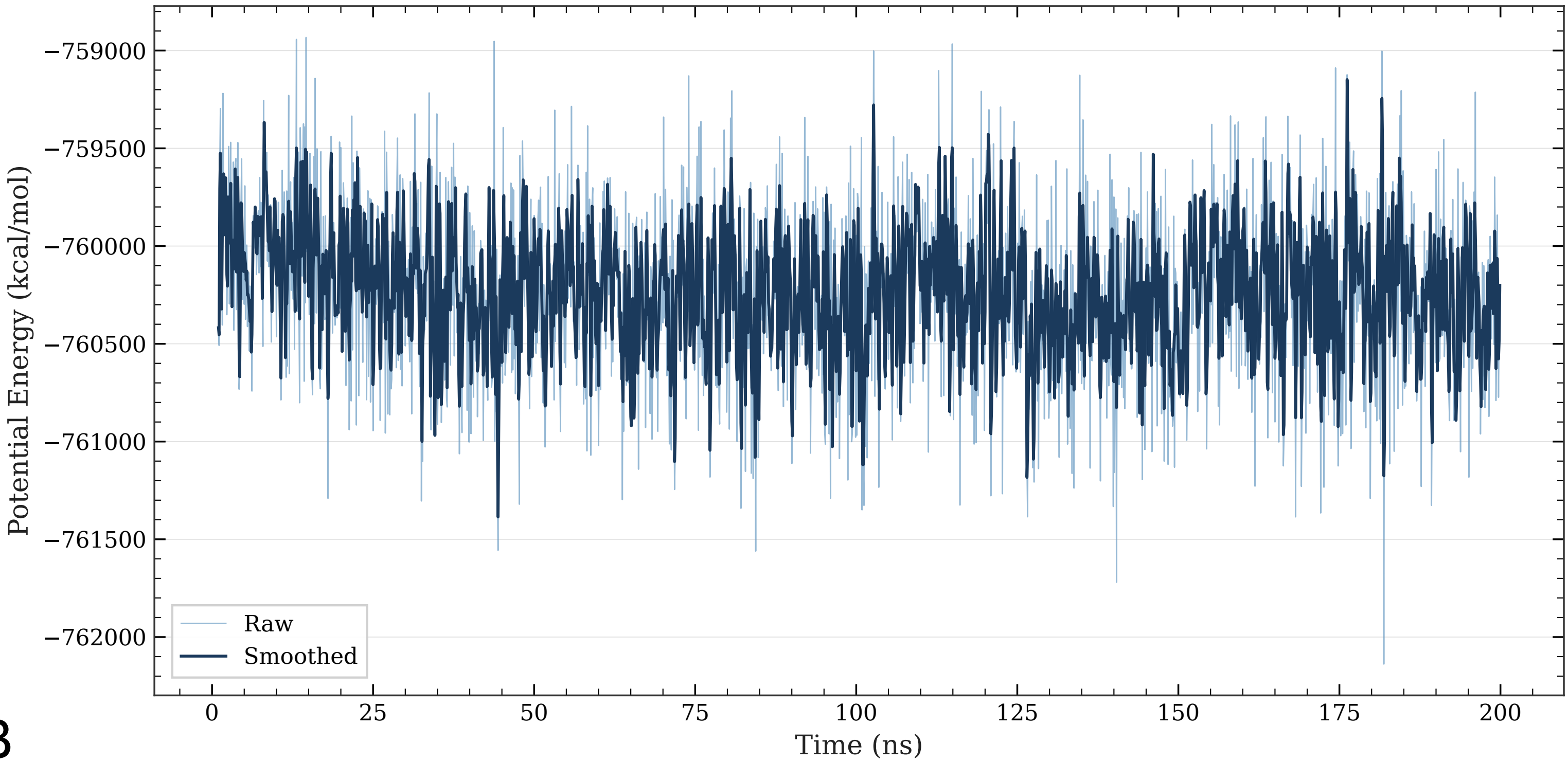

−759000
−759500
−760000
−760500
−761000
−761500
−762000
Potential Energy (kcal/mol)
0
25
50
75
100
125
150
175
200
Time (ns)
Raw
Smoothed


B

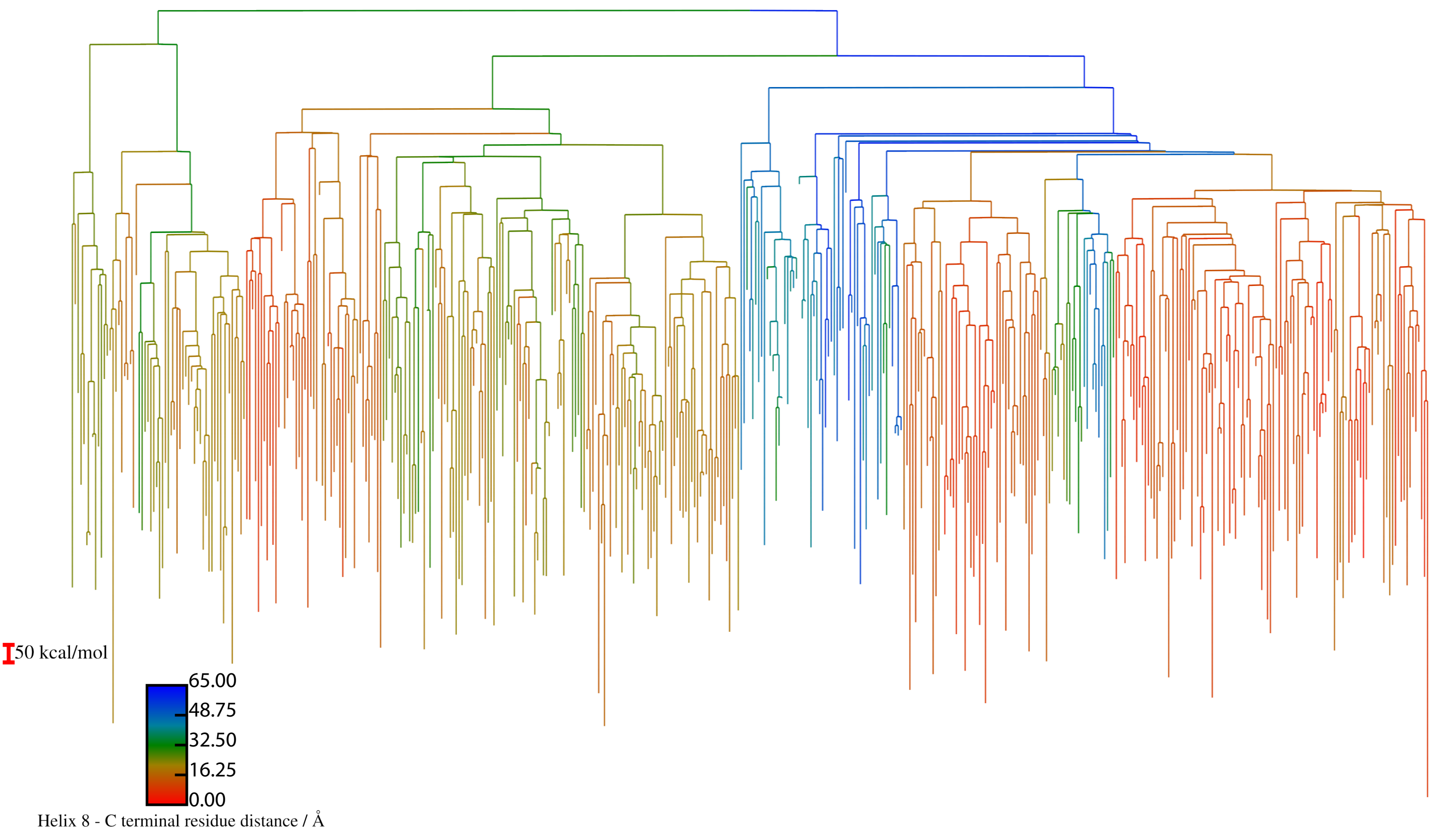

50 kcal/mol
65.00
48.75
32.50
16.25
0.00
Helix 8 - C terminal residue distance / Å

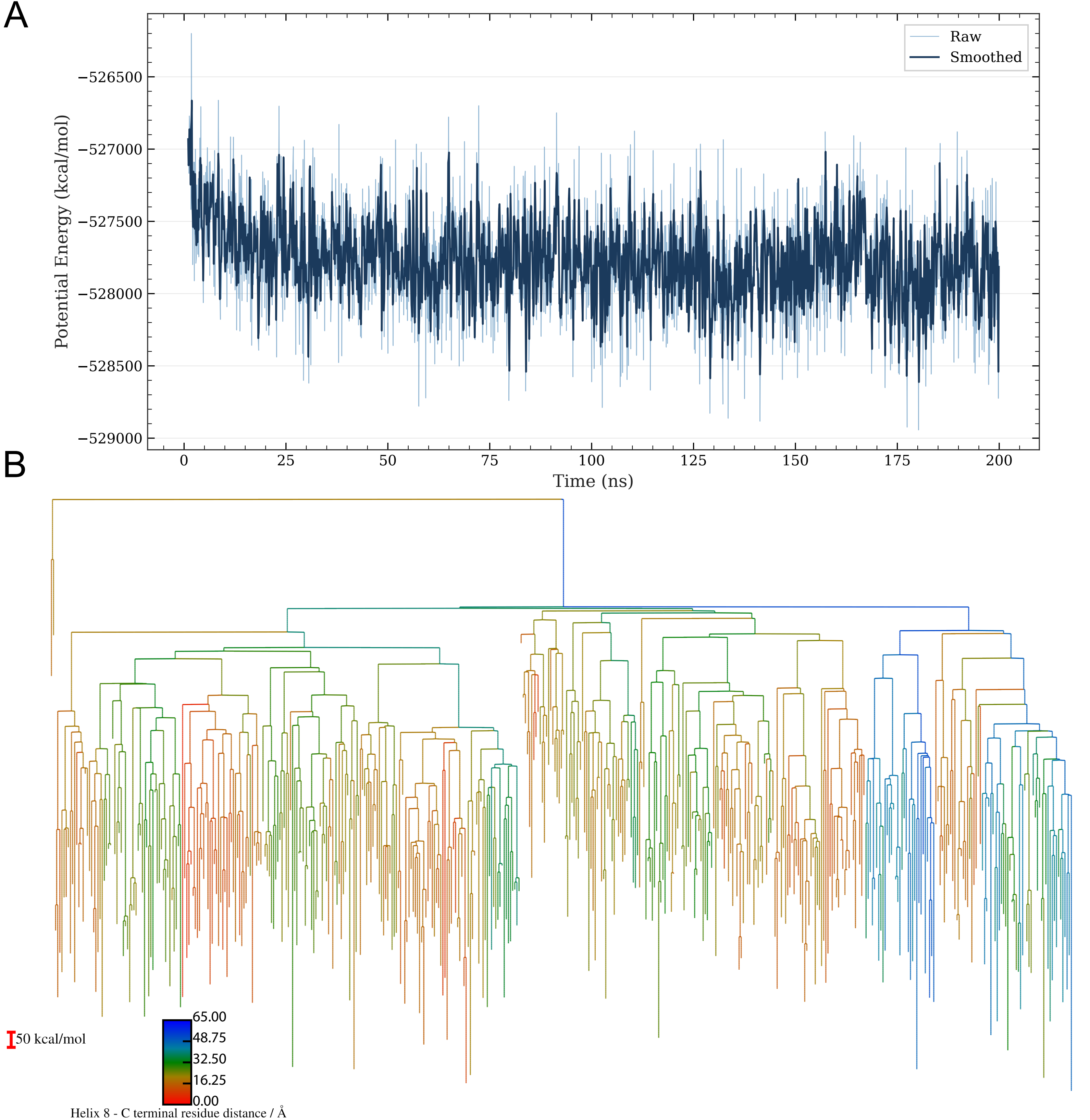
A
Raw
Smoothed
Potential Energy (kcal/mol)
−526500
−527000
−527500
−528000
−528500
−529000
0
25
50
75
100
125
150
175
200
Time (ns)
B
50 kcal/mol
65.00
48.75
32.50
16.25
0.00
Helix 8 - C terminal residue distance / Å

A

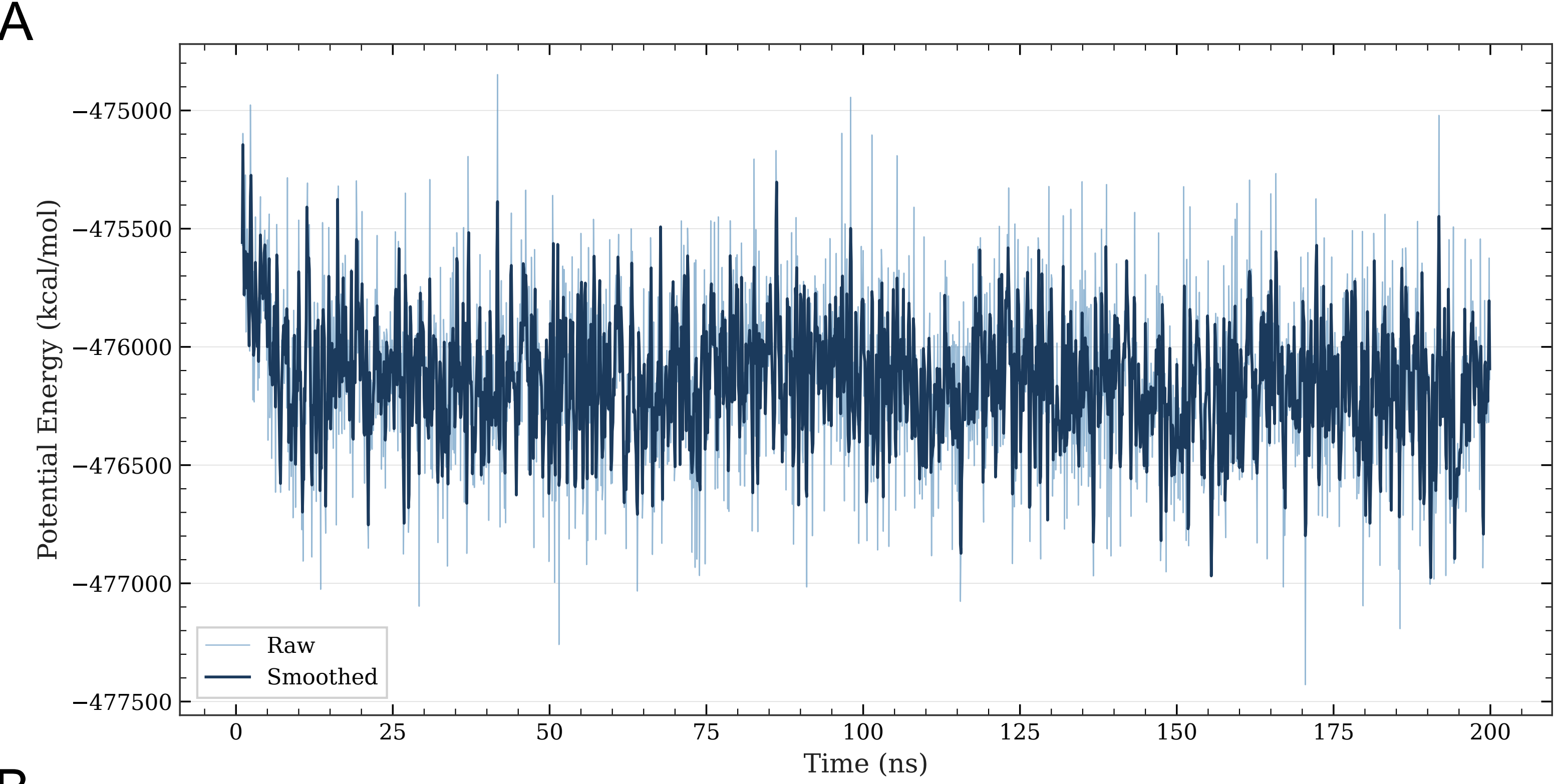

Raw
Smoothed
Potential Energy (kcal/mol)
Time (ns)
−475000
−475500
−476000
−476500
−477000
−477500
0
25
50
75
100
125
150
175
200


B

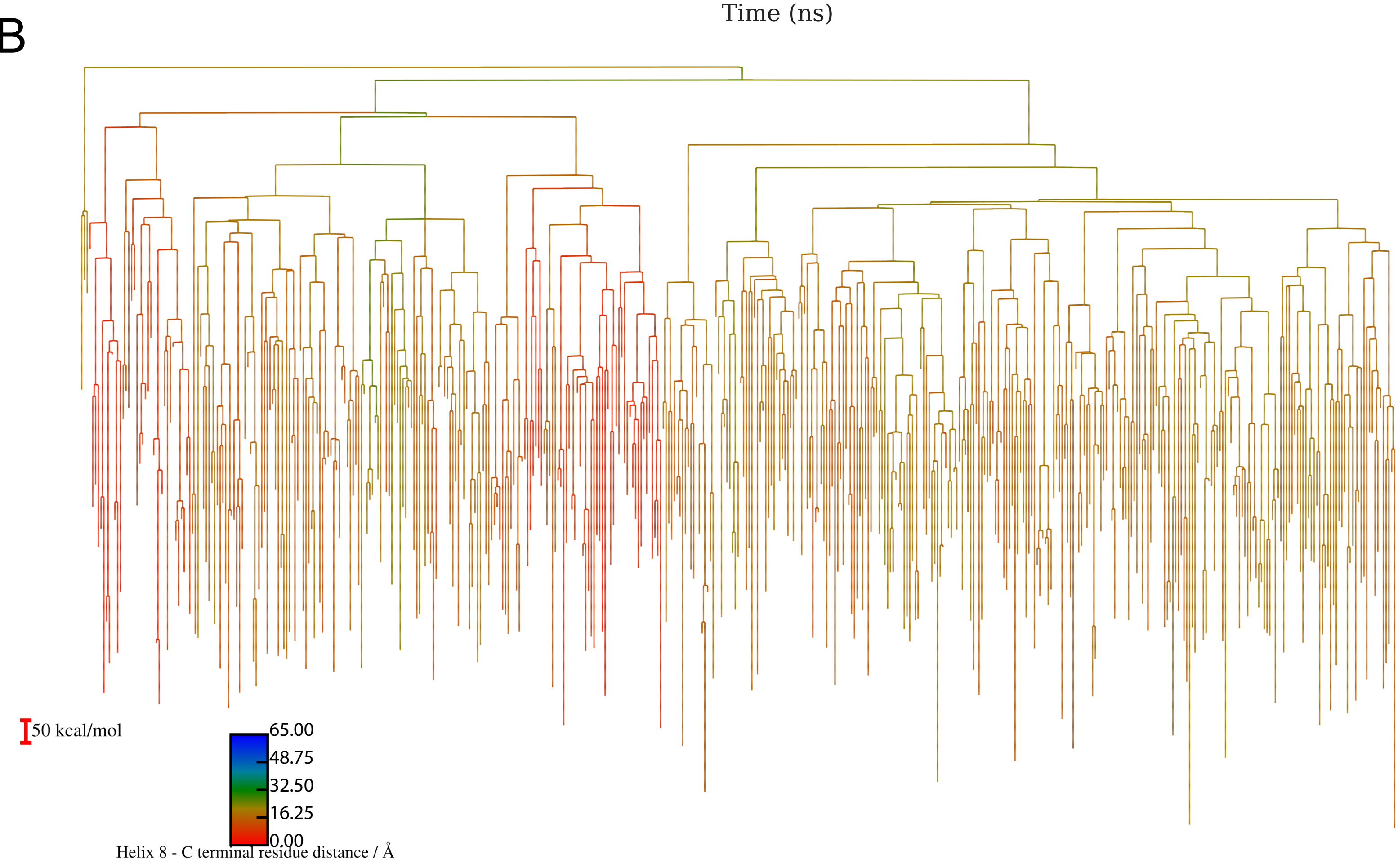

50 kcal/mol
65.00
48.75
32.50
16.25
0.00
Helix 8 - C terminal residue distance / Å

A

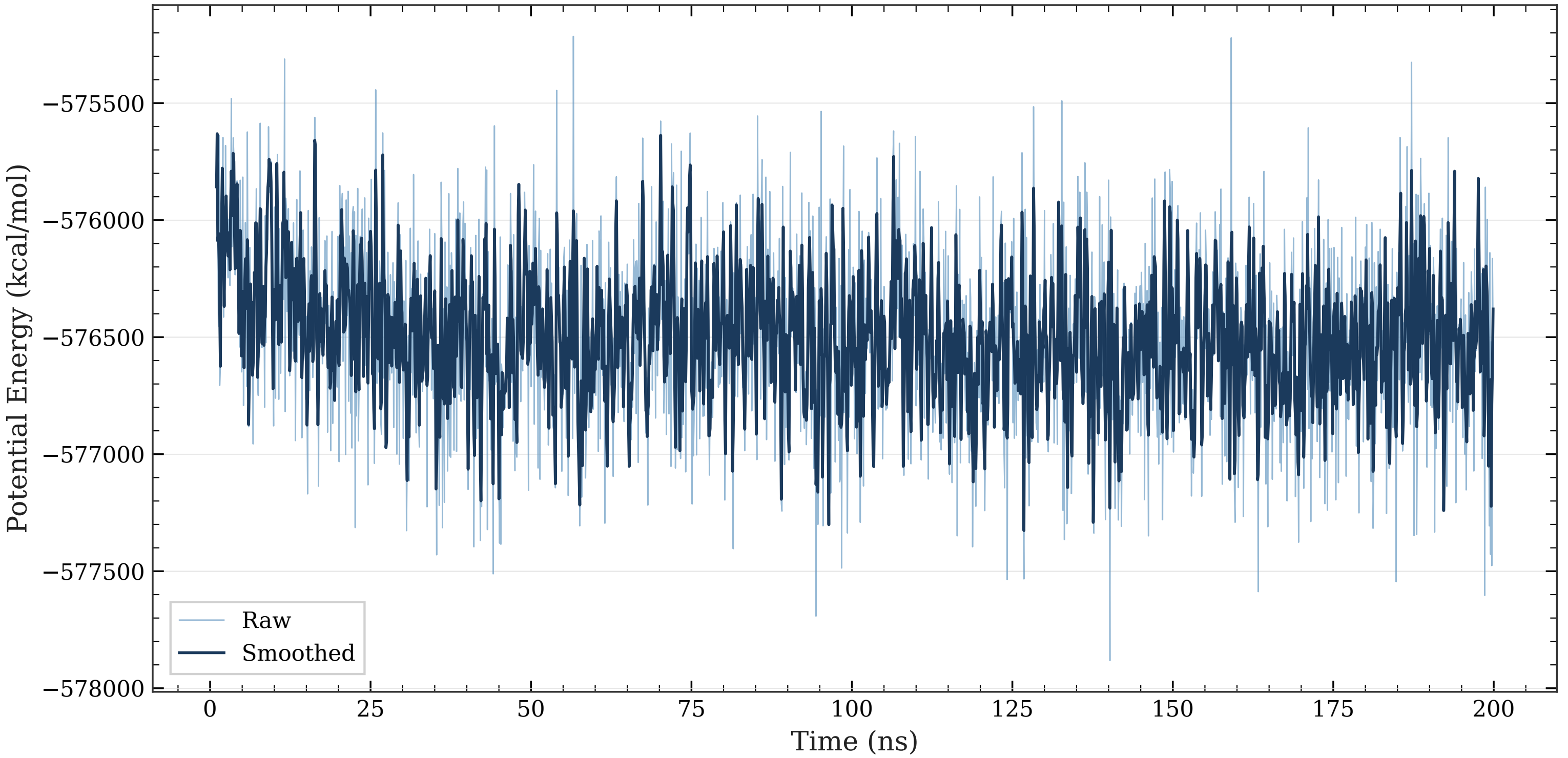

−575500
−576000
−576500
−577000
−577500
−578000
Potential Energy (kcal/mol)
0
25
50
75
100
125
150
175
200
Time (ns)
Raw
Smoothed


B

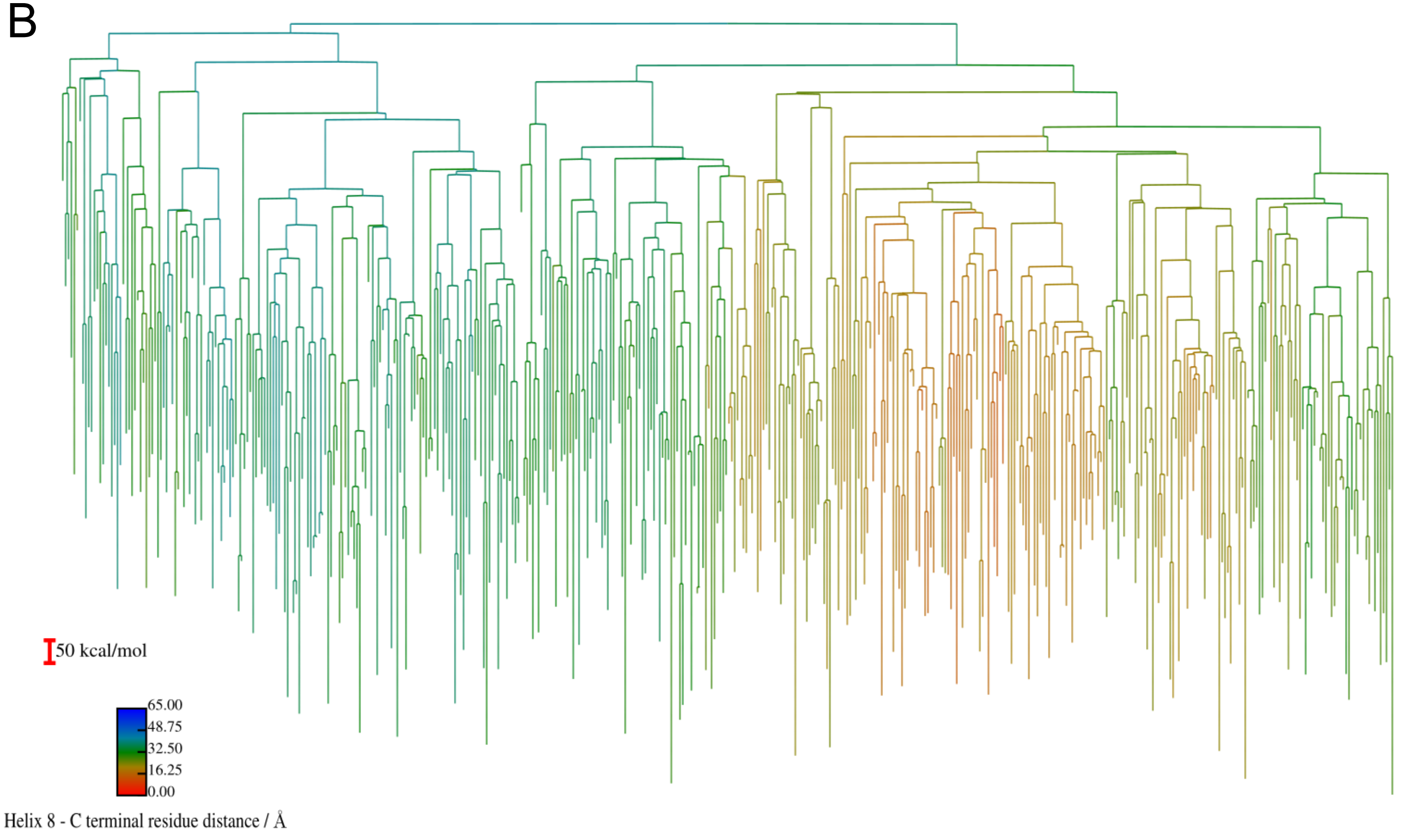

50 kcal/mol
65.00
48.75
32.50
16.25
0.00
Helix 8 - C terminal residue distance / Å

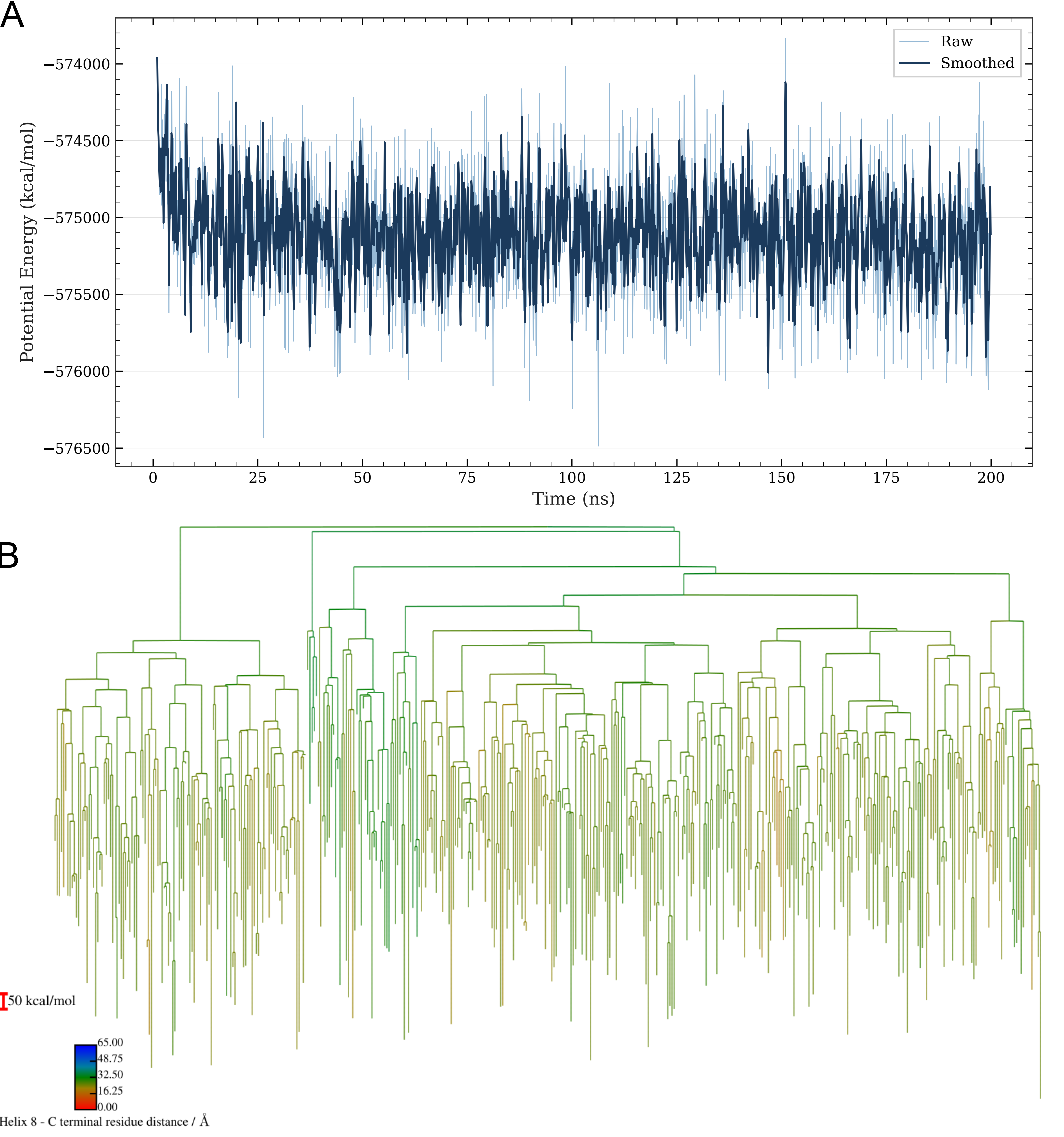
A
Raw
Smoothed
Potential Energy (kcal/mol)
−574000
−574500
−575000
−575500
−576000
−576500
0
25
50
75
100
125
150
175
200
Time (ns)
B
50 kcal/mol
65.00
48.75
32.50
16.25
0.00
Helix 8 - C terminal residue distance / Å

**Supplementary Figure 8-13. A338V alters the distribution along the C-terminal extension coordinate, indicating a mutation-driven reweighting of compact and extended conformational basins.** Disconnectivity graphs constructed from representative MD trajectories for each simulated system, shown together with the corresponding potential energy traces. The graphs are colored according to an order parameter defined as the distance between the center of the vector extending from helix 8 to the end of the C-terminal tail. This metric reflects the degree of C-terminal extension, allowing compact and extended conformations sampled along the trajectories to be distinguished**. Supplementary Figure 8.** Active GPR183 WT, **Supplementary Figure 9**. Active GPR183 A338V mutant, **Supplementary Figure 10**. Inactive GPR183 WT, **Supplementary Figure 11**. Inactive GPR183 A338V mutant, **Supplementary Figure 12**. GPR183-Nluc wild type, **Supplementary Figure 13**. GPR183-Nluc A338V.

## References

(1) Chen, H.; Huang, W.; Li, X. Structures of oxysterol sensor EBI2/GPR183, a key regulator of the immune response. *Structure* **2022**, *30* (7), 1016-1024 e1015. DOI: 10.1016/j.str.2022.04.006.
(2) Abramson, J.; Adler, J.; Dunger, J.; Evans, R.; Green, T.; Pritzel, A.; Ronneberger, O.; Willmore, L.; Ballard, A. J.; Bambrick, J.; et al. Accurate structure prediction of biomolecular interactions with AlphaFold 3. *Nature* **2024**, *630* (8016), 493-500. DOI: 10.1038/s41586-024-07487-w.
(3) Fiser, A.; Sali, A. ModLoop: automated modeling of loops in protein structures. *Bioinformatics* **2003**, *19* (18), 2500-2501. DOI: 10.1093/bioinformatics/btg362.
(4) Mirdita, M.; Schutze, K.; Moriwaki, Y.; Heo, L.; Ovchinnikov, S.; Steinegger, M. ColabFold: making protein folding accessible to all. *Nat Methods* **2022**, *19* (6), 679-682. DOI: 10.1038/s41592-022-01488-1.
(5) Pieper, U.; Webb, B. M.; Dong, G. Q.; Schneidman-Duhovny, D.; Fan, H.; Kim, S. J.; Khuri, N.; Spill, Y. G.; Weinkam, P.; Hammel, M.; et al. ModBase, a database of annotated comparative protein structure models and associated resources. *Nucleic Acids Res* **2014**, *42* (Database issue), D336-346. DOI: 10.1093/nar/gkt1144.
(6) Wang, J.; Wolf, R. M.; Caldwell, J. W.; Kollman, P. A.; Case, D. A. Development and testing of a general amber force field. *J Comput Chem* **2004**, *25* (9), 1157-1174. DOI: 10.1002/jcc.20035.
(7) Schott-Verdugo, S.; Gohlke, H. PACKMOL-Memgen: A Simple-To-Use, Generalized Workflow for Membrane-Protein-Lipid-Bilayer System Building. *Journal of chemical information and modeling* **2019**, *59* (6), 2522-2528. DOI: 10.1021/acs.jcim.9b00269.
(8) Mark, P.; Nilsson, L. Structure and dynamics of the TIP3P, SPC, and SPC/E water models at 298 K. *Journal of Physical Chemistry B* **2001**, *105* (43), 24a-24a.
(9) Maier, J. A.; Martinez, C.; Kasavajhala, K.; Wickstrom, L.; Hauser, K. E.; Simmerling, C. ff14SB: Improving the Accuracy of Protein Side Chain and Backbone Parameters from ff99SB. *J Chem Theory Comput* **2015**, *11* (8), 3696-3713. DOI: 10.1021/acs.jctc.5b00255.
(10) Dickson, C. J.; Madej, B. D.; Skjevik, A. A.; Betz, R. M.; Teigen, K.; Gould, I. R.; Walker, R. C. Lipid14: The Amber Lipid Force Field. *J Chem Theory Comput* **2014**, *10* (2), 865-879. DOI: 10.1021/ct4010307.
(11) Ciura, P.; Smardz, P.; Spodzieja, M.; Sieradzan, A. K.; Krupa, P. Multilayered Computational Framework for Designing Peptide Inhibitors of HVEM-LIGHT Interaction. *The journal of physical chemistry. B* **2024**, *128* (28), 6770-6785. DOI: 10.1021/acs.jpcb.4c02255.
(12) Roe, D. R.; Cheatham, T. E., 3rd. PTRAJ and CPPTRAJ: Software for Processing and Analysis of Molecular Dynamics Trajectory Data. *J Chem Theory Comput* **2013**, *9* (7), 3084-3095. DOI: 10.1021/ct400341p.
(13) Neuman, V.; Wesolowski, P. A.; Bojarski, K. K.; Dewan, D.; Schaffler, M.; Smardz, P.; Wales, D. J. Visualizing the energy landscape for a molecular dynamics trajectory. *J Chem Phys* **2026**, *164* (4). DOI: 10.1063/5.0310206.
(14) Becker, O. M.; Karplus, M. The topology of multidimensional potential energy surfaces: Theory and application to peptide structure and kinetics. *J Chem Phys* **1997**, *106* (4), 1495-1517. DOI: Doi 10.1063/1.473299. Wales, D. J.; Miller, M. A.; Walsh, T. R. Archetypal energy landscapes. *Nature* **1998**, *394* (6695), 758-760. DOI: Doi 10.1038/29487.

(15) Savitzky, A.; Golay, M. J. E. Smoothing + Differentiation of Data by Simplified Least Squares Procedures. *Analytical chemistry* **1964**, *36* (8), 1627-&. DOI: DOI 10.1021/ac60214a047.
(16) Nerin-Fonz, F.; Caprai, C.; Morales-Pastor, A.; Lopez-Balastegui, M.; Aranda-Garcia, D.; Giorgino, T.; Selent, J. AlloViz: A tool for the calculation and visualisation of protein allosteric communication networks. *Comput Struct Biotechnol J* **2024**, *23*, 1938-1944. DOI: 10.1016/j.csbj.2024.04.047.
(17) Dror, R. O.; Arlow, D. H.; Maragakis, P.; Mildorf, T. J.; Pan, A. C.; Xu, H.; Borhani, D. W.; Shaw, D. E. Activation mechanism of the beta2-adrenergic receptor. *Proc Natl Acad Sci U S A* **2011**, *108* (46), 18684-18689. DOI: 10.1073/pnas.1110499108.
(18) Wright, S. C.; Kozielewicz, P.; Kowalski-Jahn, M.; Petersen, J.; Bowin, C. F.; Slodkowicz, G.; Marti-Solano, M.; Rodriguez, D.; Hot, B.; Okashah, N.; et al. A conserved molecular switch in Class F receptors regulates receptor activation and pathway selection. *Nature communications* **2019**, *10* (1), 667. DOI: 10.1038/s41467-019-08630-2.
(19) Andersson, L.; Roggia, M.; Wangriatisak, K.; Kozel, R. S.; Brittain, H. R.; Youhanna, S.; Gil, M.; Haag, M.; Lauschke, V. M.; Chemin, K.; et al. Structure-based screening and a conformational biosensor identify a GPR183 inverse agonist and an activation switch. *Nature communications* **2026**, *17* (1). DOI: 10.1038/s41467-026-73857-9.
(20) Schihada, H.; Kowalski-Jahn, M.; Turku, A.; Schulte, G. Deconvolution of WNT-induced Frizzled conformational dynamics with fluorescent biosensors. *Biosens Bioelectron* **2021**, *177*, 112948. DOI: 10.1016/j.bios.2020.112948.
(21) Klarenbeek, J.; Goedhart, J.; van Batenburg, A.; Groenewald, D.; Jalink, K. Fourth-generation epac-based FRET sensors for cAMP feature exceptional brightness, photostability and dynamic range: characterization of dedicated sensors for FLIM, for ratiometry and with high affinity. *PLoS One* **2015**, *10* (4), e0122513. DOI: 10.1371/journal.pone.0122513.
